# Waveband-resolved nonlinear characterization of SERF-magnetocardiography signals: a phase-space reconstruction framework for exploratory clinical cohort analysis

**DOI:** 10.3389/fphys.2026.1770392

**Published:** 2026-09-17

**Authors:** Keyi Li, Xiangyang Zhou, Jiaojiao Pang, Rui Shang, Yadan Zhang, Yangyang Cui, Dong Xu, Min Xiang

**Affiliations:** 1 Key Laboratory of Ultra-Weak Magnetic Field Measurement Technology, Ministry of Education, School of Instrumentation and Optoelectronic Engineering, Beihang University, Beijing, China; 2 Zhejiang Provincial Key Laboratory of Ultra-Weak Magnetic-Field Space and Applied Technology, Hangzhou Innovation Institute, Beihang University, Hangzhou, China; 3 Hefei National Laboratory, Hefei, China; 4 Department of Emergency Medicine, Qilu Hospital of Shandong University, Jinan, China; 5 Shandong Key Laboratory for Magnetic Field-free Medicine & Functional Imaging, National Medicine-Engineering Interdisciplinary Industry-Education Integration Innovation Platform, Qilu Hospital of Shandong University, Jinan, China; 6 Shandong Provincial Clinical Research Center for Emergency and Critical Care Medicine, Institute of Emergency and Critical Care Medicine of Shandong University, Chest Pain Center,Qilu Hospital of Shandong University, Jinan, China; 7 National Medical Products Administration (NMPA) Key Laboratory for Clinical Research and Evaluation of Innovative Drug, Qilu Hospital of Shandong University, Jinan, China; 8 Hangzhou Institute of National Extremely-Weak Magnetic Field Infrastructure, Hangzhou, China; 9 State Key Laboratory of Traditional Chinese Medicine Syndrome/National Institute of Extremely-Weak Magnetic Field Infrastructure, Hangzhou, China

**Keywords:** magnetocardiography, SERF magnetometer, nonlinear dynamics, phase-space reconstruction, self-attention, exploratory signal characterization, cardiac magnetic fields, chaos theory

## Abstract

**Background:**

Spin-exchange relaxation-free magnetocardiography (SERF–MCG) offers femtotesla-level sensitivity for noninvasive cardiac monitoring. Conventional analysis pipelines, largely adapted from linear electrocardiography, may not fully capture nonlinear dynamical patterns embedded in cardiac magnetic-field signals. In this retrospective and de-identified cohort study, we aimed to develop and evaluate a waveband-resolved nonlinear analysis framework for exploratory signal-level characterization of SERF–MCG recordings from stringently screened healthy adults and a heterogeneous clinical cardiovascular patient cohort enriched for clinically diagnosed ischemia.

**Methods:**

We developed a waveband-resolved framework that decomposes each SERF–MCG beat into P, QRS, ST, and T segments and reconstructs segment-specific attractors via delay-coordinate embedding. From these reconstructed trajectories, we extracted a 32-dimensional feature set capturing phase-space geometric descriptors, entropy-related descriptors, Lyapunov-related descriptors, and spatial energy/eigenvalue-ratio descriptors. Cross-phase signal associations were assessed using a dual-perspective approach: (i) statistical association matrices derived from feature aggregates and (ii) a four-node self-attention encoder that estimates a 4 × 4 matrix of model-derived association weights between cardiac phases.

**Results:**

Analysis of 1,848 statistically independent median beats, including 906 stringently screened healthy controls and 942 recordings from a heterogeneous clinical cardiovascular patient cohort enriched for clinically diagnosed ischemia, showed statistically significant but partially overlapping nonlinear feature differences between groups. Repolarization-related ST/T features showed lower sample entropy and altered Lyapunov-related descriptors in the clinical patient cohort, consistent with group-level differences in signal complexity and local trajectory instability. Statistical and attention-based analyses further suggested altered ST–T association patterns at the signal-feature level. These findings should be interpreted as exploratory cohort-level signal differences rather than ischemia-specific causal or diagnostic evidence.

**Conclusions:**

This study presents a waveband-resolved nonlinear SERF–MCG analysis framework for exploratory characterization of cardiac magnetic-field signals. Within the present retrospective and de-identified dataset, the framework identified group-level differences in repolarization-related nonlinear features and cross-phase association patterns between stringently screened healthy controls and a heterogeneous clinical patient cohort. The findings support the methodological feasibility of phase-space reconstruction and attention-assisted association analysis for SERF–MCG signal characterization, while prospective studies with complete clinical covariates and uniform diagnostic verification are required before ischemia-specific or diagnostic claims can be made.

## 1 Introduction

Cardiovascular disease can alter cardiac electrophysiology before overt morphological abnormalities become clearly detectable on conventional surface electrocardiography (ECG). These alterations may involve changes in action-potential duration, conduction, repolarization stability, and the spatiotemporal coordination of cardiac activation and recovery. Standard 12-lead ECG remains the primary clinical tool for cardiac assessment, but its sensitivity to subtle or spatially complex electrophysiological changes may be limited by the attenuation and projection of electrical signals through thoracic tissues. In contrast, magnetocardiography (MCG) records the magnetic fields generated by intramyocardial currents and may provide complementary information about cardiac electromagnetic activity Fenici et al. (2005); Kominis et al. (2003).

Recent technological breakthroughs in spin-exchange relaxation-free (SERF) magnetometers Kominis et al. (2003); Budker and Romalis (2007) have enabled the development of compact, cryogen-free MCG arrays with femtotesla-level sensitivity Kominis et al. (2003); Budker and Romalis (2007); Boto et al. (2018). These hardware advances have renewed interest in MCG-based cardiac signal analysis, including morphology-based markers, nonlinear signal descriptors, and modern machine learning approaches. Parallel progress in AI-enabled ECG has demonstrated that deep learning can detect occlusive myocardial infarction (OMI) even when classical criteria are nondiagnostic Attia et al. (2019); Al-Zaiti et al. (2023). However, most existing analytical methods treat cardiac signals as linear time series, focusing primarily on gross morphology or global time-frequency features. Such approaches risk overlooking the complex, nonlinear dynamical feedback that governs cardiac stability.

From a dynamical systems perspective, the heart is a nonlinear oscillator exhibiting chaos, bifurcations, and complex attractor geometry, particularly under pathological conditions Goldberger et al. (2002); Voss et al. (2009); Costa et al. (2002). Techniques derived from nonlinear dynamics—including phase-space reconstruction (PSR), entropy measures, and Lyapunov exponents—have provided profound insights into heart rate variability and arrhythmogenesis Goldberger et al. (2002); Voss et al. (2009); Costa et al. (2002). Yet, these tools have rarely been applied systematically to MCG, and almost never in a *waveband-resolved* manner that explicitly separates atrial depolarization (P wave), ventricular depolarization (QRS complex), early repolarization (ST segment), and late repolarization (T wave).

From a physiological perspective, repolarization-related wavebands such as the ST segment and T wave are of particular interest because they reflect phases of ventricular recovery that may be affected by metabolic, structural, or electrophysiological stress, including ischemia-related alterations in repolarization reserve and restitution dynamics Heusch (2020); Antzelevitch and Fish (2001). However, in a retrospective and de-identified dataset without complete individual-level clinical covariates or uniform diagnostic verification, observed signal differences cannot be attributed uniquely to myocardial ischemia. Therefore, in the present study, we treat waveband-resolved nonlinear features as exploratory signal-level descriptors that may characterize differences between healthy reference recordings and heterogeneous clinical patient recordings, rather than as definitive ischemia-specific biomarkers.

In this study, we analyze SERF-MCG beats as phase-resolved nonlinear dynamical processes, treating P, QRS, ST, and T segments as distinct yet coupled components of the cardiac cycle. Our primary goal is not to establish a definitive diagnostic classifier, but to evaluate whether a waveband-resolved nonlinear framework can provide interpretable signal-level descriptors that distinguish stringently screened healthy controls from a heterogeneous clinical cardiovascular patient cohort.

Our main contributions are as follows:

1. We introduce a waveband-specific PSR pipeline for SERF–MCG that reconstructs the attractor geometry of P, QRS, ST, and T segments and extracts a 32-dimensional nonlinear feature set capturing trajectory geometry, entropy-related descriptors, Lyapunov-related descriptors, spatial energy, and eigenvalue-ratio structure.
2. We propose a supervised four-node self-attention encoder over waveband-level representations, yielding a 4 × 4 matrix of label-conditioned model-derived association weights among cardiac phases. These weights are used for exploratory interpretation of group-discriminative waveband relationships rather than as direct physiological coupling or causal information-flow measures.
3. We quantify cross-waveband signal associations using both correlation-based measures and attention-derived weights, performing statistical testing to characterize group-level differences in ST/T association patterns.
4. We conduct waveband ablation experiments, exploratory dimensionality reduction, and methodological sensitivity analyses to assess the contribution and limitations of repolarization-related nonlinear features in this retrospective cohort.

Large-cohort exploratory analysis. Unlike many pilot MCG studies relying on small sample sizes, this work analyzes statistically independent median beats from a large retrospective dataset of 1,848 subjects, including 906 stringently screened healthy controls and 942 heterogeneous clinical cardiovascular patient recordings enriched for clinically diagnosed ischemia. This design reduces within-subject pseudo-replication and supports a population-level exploratory analysis of SERF–MCG nonlinear features. However, because individual-level clinical covariates and uniform diagnostic verification were unavailable after de-identification, the study is framed as methodological feasibility and exploratory signal characterization rather than as ischemia-specific diagnostic validation.

A conceptual illustration of the hypothesized waveband coupling relationships is shown in **Figure 1**.

**Figure 1:**
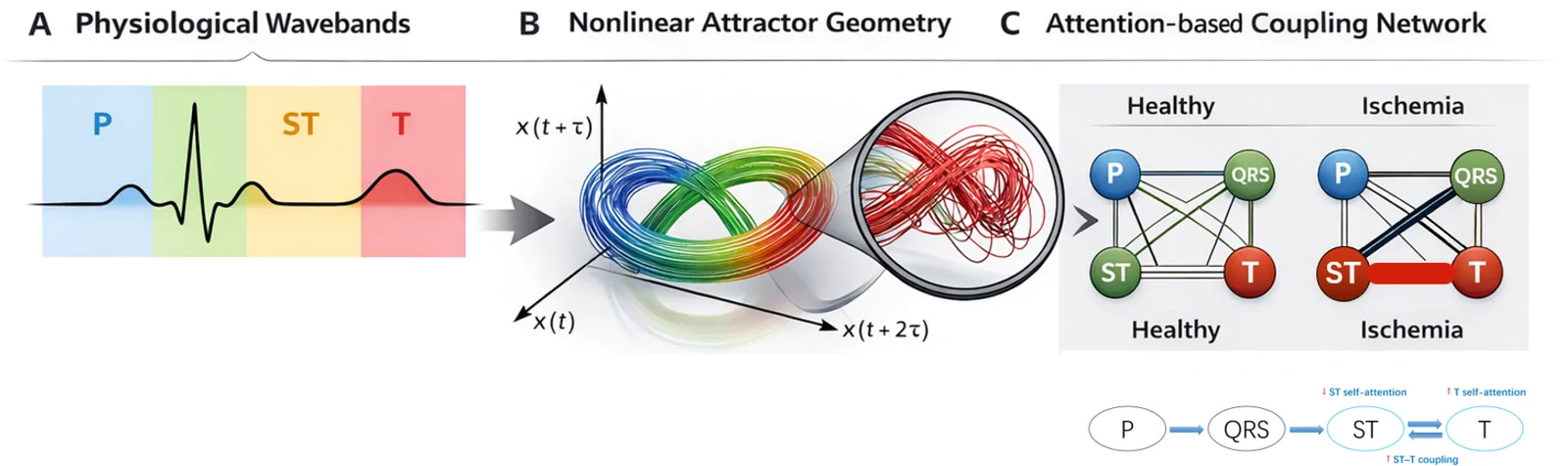
Conceptual schematic of waveband-resolved cardiac dynamics and cross-phase association analysis. **(A)** Representative cardiac waveform segmented into physiologically defined wavebands. **(B)** Phase-space representation illustrating nonlinear attractor geometry. **(C)** Schematic cross-phase association network among P, QRS, ST, and T wavebands. The schematic illustrates the working hypothesis that repolarization-related wavebands may show altered signal-level association patterns in heterogeneous clinical cardiovascular recordings compared with stringently screened healthy reference recordings. These relationships are evaluated using correlation-based and attention-derived association analyses and are interpreted as exploratory signal-level patterns rather than ischemia-specific causal mechanisms.

## 2 Materials and methods

The proposed analysis pipeline, encompassing data acquisition, waveband segmentation, phase-space reconstruction (PSR), and coupling analysis, is illustrated in **Figure 2**.

**Figure 2:**
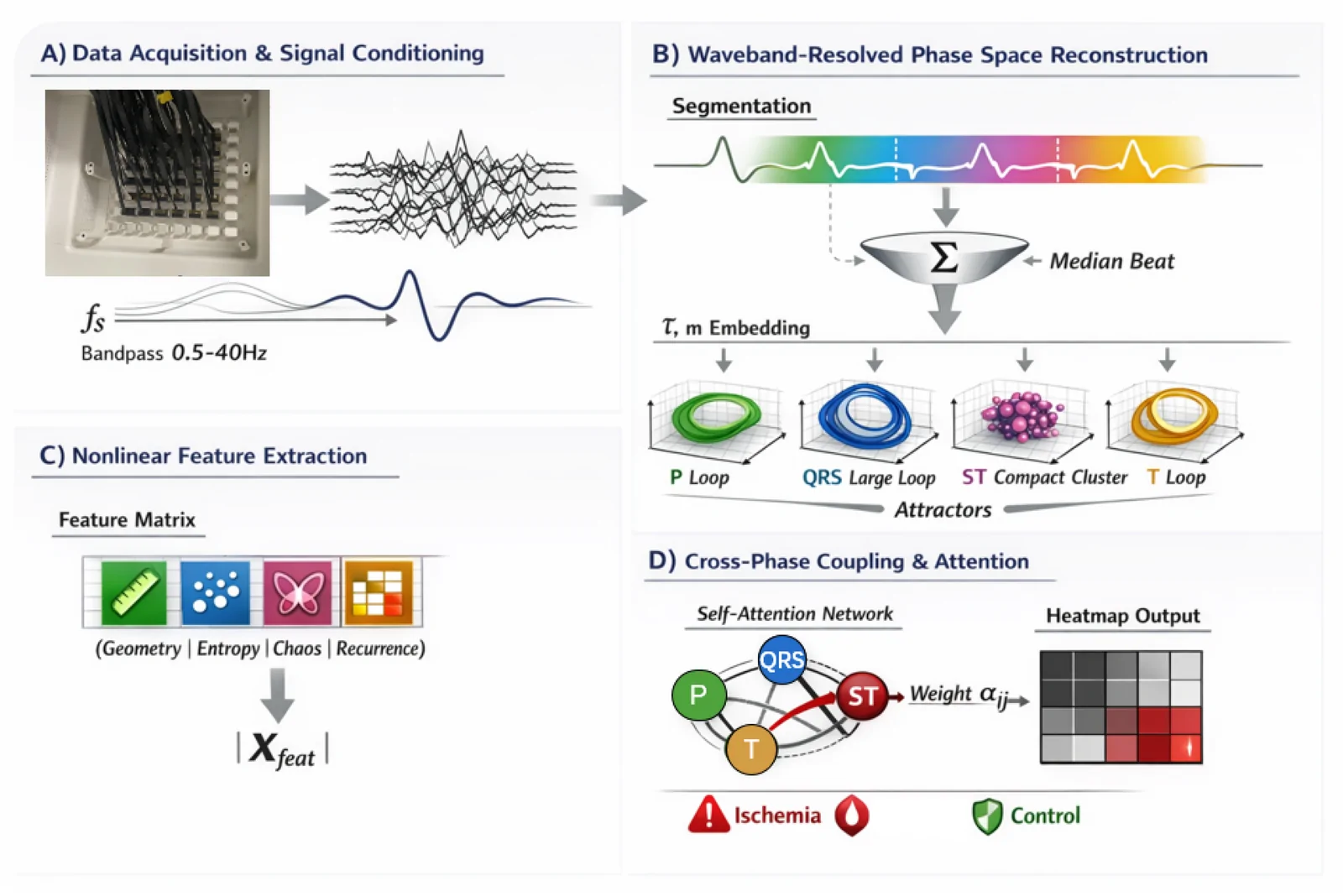
The proposed waveband-resolved nonlinear dynamics analysis framework. **(A)** Data Acquisition & Preprocessing: Thirty-six-channel SERF–MCG signals are bandpass filtered (0.5–40 Hz) and directly averaged across all channels to construct a virtual lead, improving the signal-to-noise ratio and cross-subject comparability. **(B)** Waveband-Resolved Phase-Space Reconstruction (PSR): The median beat is physiologically segmented into P, QRS, ST, and T wavebands. Subject-level embedding parameters (*τ,m*) are estimated from the representative median beat and then applied consistently to the waveband-resolved trajectories. **(C)** Nonlinear Feature Extraction: Nonlinear descriptors spanning phase-space geometry, entropy-related descriptors, Lyapunov-related descriptors, spatial energy, and eigenvalue-ratio structure are computed across the four wavebands to form an assembled feature vector **X***_feat_*. **(D)** Cross-Phase Coupling & Attention: Correlation-based measures and self-attention weights are used to summarize statistical and model-derived associations among cardiac phases. The overall framework is intended for exploratory signal-level characterization of group differences between healthy reference recordings and heterogeneous clinical patient recordings, rather than for definitive ischemia-specific diagnosis. Because the attention module is trained with cohort labels, attention-derived association patterns are interpreted as label-conditioned model-derived signal markers rather than direct evidence of intrinsic physiological coupling or causal influence.

### 2.1 Study population and clinical characterization

This retrospective study was based on a multi-year SERF–MCG database collected between 2021 and 2025. The initial dataset consisted of 2,000 independent recordings. After signal-quality control, including the exclusion of recordings with excessive motion artifacts, unstable baselines, or unreliable fiducial-point localization, 1,848 subjects were retained for analysis, including 906 stringently screened healthy controls and 942 recordings from a heterogeneous clinical cardiovascular patient cohort enriched for clinically diagnosed myocardial ischemia.

Healthy control cohort. Healthy reference individuals were selected according to the WS/T 402–2012 standard and previous clinical reference-population studies, with the aim of including only apparently healthy adults who were confirmed to be free of major cardiovascular conditions by questionnaire-based screening and subsequent clinical examinations. The inclusion criteria were: (1) age ≥ 18 years; (2) no hospitalization for any reason within the preceding 6 months; (3) no current use of prescription medications; (4) no history of cardiovascular disease, including coronary artery disease, structural heart disease, arrhythmia, heart failure, stroke, pulmonary embolism, aortic dissection, or peripheral arterial disease; (5) no cardiovascular risk factors, including hypertension, diabetes mellitus, or hyperlipidemia; (6) no smoking history or smoking cessation for more than 6 months; (7) no excessive alcohol consumption, defined as an average daily alcohol intake of less than 3 drinks; and (8) written informed consent.

The exclusion criteria for healthy controls were: (1) body mass index (BMI) *>* 28 kg/m^2^ or *<* 18 kg/m^2^; (2) resting heart rate *<* 50 or *>* 110 beats/min; (3) resting systolic blood pressure ≥ 140 mmHg and/or diastolic blood pressure ≥ 90 mmHg; (4) fasting glucose ≥ 7.0 mmol/L and/or random glucose ≥ 11.1 mmol/L; (5) total cholesterol ≥ 6.2 mmol/L or low-density lipoprotein cholesterol ≥ 4.1 mmol/L; (6) abnormal electrocardiographic findings, such as arrhythmia, ST–T abnormalities, or pathological axis deviation; (7) pathological echocardiographic findings, such as left ventricular hypertrophy, left ventricular dilation, regional wall-motion abnormalities, or more-than-mild valvular regurgitation or stenosis; (8) respiratory, hepatic, renal, endocrine, hematologic, connective-tissue, malignant, or other systemic diseases; (9) professional athlete status, pregnancy or lactation, depression, or other psychological or psychiatric disorders; and (10) inability to complete SERF–MCG examination because of claustrophobia or inability to cooperate with study procedures. Recordings were further excluded if SERF–MCG acquisition failed or if the signal quality was insufficient for reliable analysis.

Clinical cardiovascular patient cohort enriched for clinically diagnosed ischemia. The clinical patient cohort consisted of real-world patients recruited from Qilu Hospital of Shandong University. Myocardial ischemia was diagnosed by experienced clinicians based on routine clinical evaluation in the hospital setting. Diagnostic information could include clinical symptoms, surface electrocardiography, coronary computed tomography angiography, invasive coronary angiography, stress-related examinations, laboratory tests, and other clinically indicated assessments, depending on each patient’s diagnostic pathway. Because this was a retrospective de-identified database, the exact diagnostic modality and timing relative to ischemic symptoms could not be reconstructed for each individual waveform. Therefore, this cohort should be interpreted as a heterogeneous clinical cardiovascular patient cohort enriched for clinically diagnosed ischemia, rather than as a uniformly angiography-confirmed ischemia cohort. Accordingly, this cohort was not used to establish ischemia-specific causal inference or diagnostic validity. Instead, it was used for exploratory signal-level comparison against the stringently screened healthy reference cohort. The lack of uniform diagnostic verification introduces potential label heterogeneity and label noise, which are explicitly considered in the interpretation and limitations of this study.

De-identification and available clinical information. During the multi-year data aggregation process, strict de-identification procedures were applied before signal analysis. As a result, individual-level demographic variables, including exact age, sex, BMI, medication status, and detailed comorbidity profiles, were irreversibly unlinked from the raw SERF–MCG waveform files and could not be reconstructed. To address the reviewer’s concern transparently, we added **Table 1**, which summarizes all available cohort-level clinical information and explicitly identifies variables that were unavailable because of de-identification. This limitation precluded propensity-score matching or covariate-adjusted modeling for demographic and medication-related confounders. However, the stringent screening of the healthy control cohort helped reduce the likelihood that the reference group contained overt cardiovascular disease, cardiovascular risk factors, medication effects, or abnormal ECG/echocardiographic findings.

**Table 1:** Available clinical characterization and de-identification transparency of the study cohorts.

Item	Healthy control cohort	Clinical cardiovascular patient cohort enriched for clinically diagnosed ischemia	Availability/interpretation
Number of analyzed subjects	906	942	Available after signal-quality control.
Data source	Healthy reference individuals screened according to WS/T 402–2012 and clinical reference-population criteria	Real-world patients recruited from Qilu Hospital of Shandong University	Available at cohort level.
Calendar period	2021–2025		Available at database level.
Age	Adults aged ≥18 years by inclusion criterion	Exact age unavailable after de-identification	Individual-level age could not be reconstructed; age matching was therefore not possible.
Sex	Unavailable after de-identification	Unavailable after de-identification	Individual-level sex information could not be reconstructed.
BMI	Restricted by exclusion criteria: BMI 18–28 kg/m^2^	Exact BMI unavailable after de-identification	Healthy controls with abnormal BMI were excluded; patient-level BMI adjustment was not possible.
Medication status	Current prescription medication use was excluded	Detailed medication records unavailable after de-identification	Medication-related confounding could not be fully adjusted in the clinical patient cohort.
Cardiovascular history	History of cardiovascular disease was excluded	Heterogeneous real-world clinical cardiovascular patients enriched for clinically diagnosed ischemia	Healthy controls were screened to exclude overt cardiovascular disease.
Cardiovascular risk factors	Hypertension, diabetes mellitus, and hyperlipidemia were excluded	Detailed risk-factor profiles unavailable after de-identification	Risk-factor adjustment in the clinical patient cohort was not possible.
Smoking and alcohol	Smoking history and excessive alcohol consumption were excluded	Unavailable after de-identification	Available only as exclusion criteria for healthy controls.
Resting heart rate	Restricted by exclusion criteria: 50–110 beats/min	Exact heart-rate distribution unavailable after de-identification	Extreme bradycardia/tachycardia was excluded from healthy controls; patient-level adjustment was not possible.
Blood pressure	Resting BP <140/90 mmHg required	Exact blood-pressure distribution unavailable after de-identification	Hypertensive-range BP was excluded from healthy controls.
Glucose and lipids	Hyperglycemia and hypercholesterolemia were excluded by predefined thresholds	Exact laboratory distributions unavailable after de-identification	Available only as exclusion criteria for healthy controls.
ECG and echocardiography	Abnormal ECG and pathological echocardiographic findings were excluded	Clinical diagnostic evaluation was performed according to routine hospital practice	Healthy controls were screened to exclude abnormal ECG/echocardiographic findings.
Clinical patient cohort definition	Not applicable	Heterogeneous real-world clinical cardiovascular patient cohort enriched for clinically diagnosed ischemia	The cohort should not be interpreted as a uniformly angiography-confirmed ischemia cohort.
Diagnostic modalities	Not applicable	Clinical symptoms, ECG, coronary CTA, invasive coronary angiography, stress-related examinations, laboratory tests, or other clinically indicated assessments may have contributed to diagnosis	Exact modality counts could not be reconstructed after de-identification.
Timing relative to ischemic event	Not applicable	Unavailable after de-identification	Acute versus chronic timing could not be reliably reconstructed.
Key limitation	Strictly screened healthy baseline	Heterogeneous clinical cardiovascular patient cohort without reconstructable individual-level covariates	Demographic matching, medication adjustment, and comorbidity adjustment could not be performed.

Because strict de-identification was applied during the multi-year retrospective aggregation of SERF–MCG data, individual-level demographic and medication variables were not available for statistical adjustment. This table summarizes the cohort-level information that was available and explicitly identifies variables that could not be reconstructed.

### 2.2 Scientific framing of the retrospective cohort

Because individual-level demographic variables, medication use, comorbidities, and uniform diagnostic verification were not reconstructable after de-identification, the present dataset cannot support ischemia-specific causal attribution or definitive diagnostic accuracy claims. The asymmetry between the stringently screened healthy control cohort and the heterogeneous clinical patient cohort may introduce spectrum bias, and the observed group differences may reflect a combination of ischemia-related, cardiovascular, medication-related, demographic, or other clinical factors.

Therefore, the scientific question was reframed as follows: whether waveband-resolved nonlinear SERF–MCG features can characterize exploratory signal-level differences between stringently screened healthy adults and a heterogeneous clinical cardiovascular patient cohort enriched for clinically diagnosed ischemia. Throughout the revised manuscript, the terms “group differences”, “clinical patient cohort”, and “exploratory signal-level characterization” are used to avoid overinterpreting the findings as ischemia-specific causal or diagnostic evidence.

### 2.3 Data acquisition and median beat extraction

SERF–MCG recordings were acquired using a 36-channel spin-exchange relaxation-free (SERF) magnetometer array Kominis et al. (2003); Budker and Romalis (2007) (**Figure 3**). For each subject, continuous SERF–MCG signals were recorded for 90 seconds at a hardware sampling rate of *f_s_*= 1000 Hz in a magnetically shielded room. All 36 channels were retained for virtual-lead construction.

**Figure 3:**
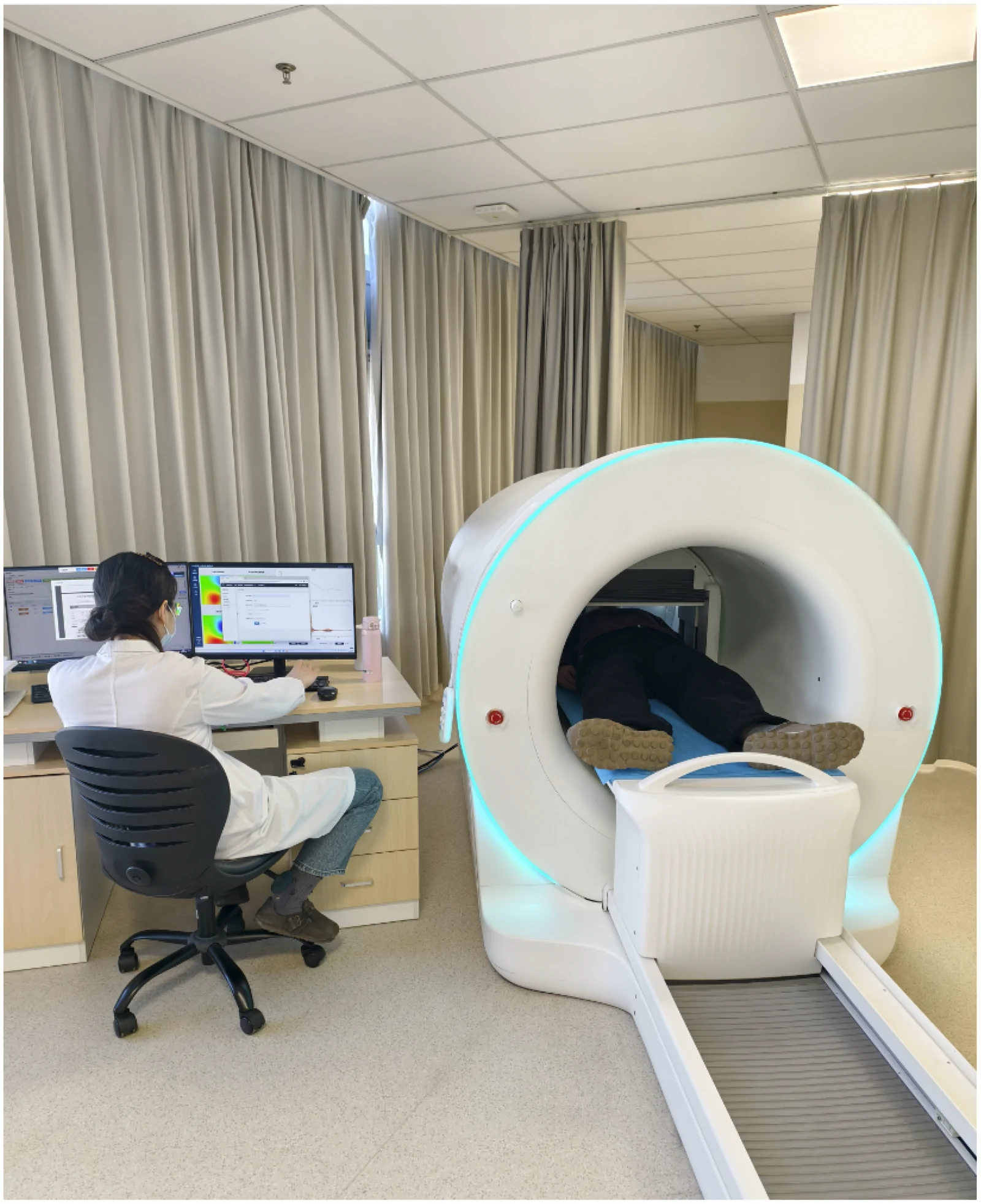
SERF–magnetocardiography (MCG) acquisition setup. The subject is positioned beneath a 36-channel SERF magnetometer array, enabling non-contact measurement of cardiac magnetic fields.

Virtual-lead Construction and Filtering. To improve signal-to-noise ratio (SNR) and cross-subject comparability, a virtual lead was constructed from the full 36-channel SERF–MCG array. Specifically, after baseline correction and filtering, the signals from all 36 channels were directly averaged point-by-point to obtain a single global virtual lead for each subject. This full-channel averaging procedure emphasizes the global cardiac magnetic-field pattern while suppressing channel-specific environmental and sensor noise.

Signals were band-pass filtered using a zero-phase filter at 0.5–40 Hz, and baseline drift was removed before beat extraction. Given the 90-second recording duration and a typical resting heart rate of approximately 70 beats/min, approximately 100–105 cardiac cycles were initially captured per subject. R-peaks were detected using the Pan–Tompkins algorithm Pan and Tompkins (1985), followed by template-based quality control. To reduce the influence of arrhythmias, ectopic beats, motion artifacts, and poorly aligned beats, a subject-specific predominant beat morphology was first estimated. Individual beats with a Pearson correlation coefficient *<* 0.90 relative to this predominant morphology were excluded. The remaining retained beats, typically 80–90 beats per subject, were aligned around the R-peak using cross-correlation maximization within a local temporal window to reduce residual phase jitter. A representative median beat was then computed point-by-point from the aligned retained beats.

This procedure was designed to ensure that one statistically independent representative beat was analyzed per subject, thereby reducing within-subject pseudo-replication while preserving the dominant morphology of the subject’s SERF–MCG waveform.

Waveband Definition. Using physiologically defined fiducial points (P-onset/offset, QRS-onset/offset, ST-onset/offset, T-onset/offset), each median beat was decomposed into four non-overlapping segments: P, QRS, ST, and T (**Figure 4**). Fiducial localization utilized a template-assisted strategy combining slope and curvature constraints. The average data length *N* for each waveband was approximately: P (∼80 pts), QRS (∼100 pts), ST (∼120 pts), and T (∼160 pts). This yields four segmental time series for each beat *n*:

**Figure 4:**
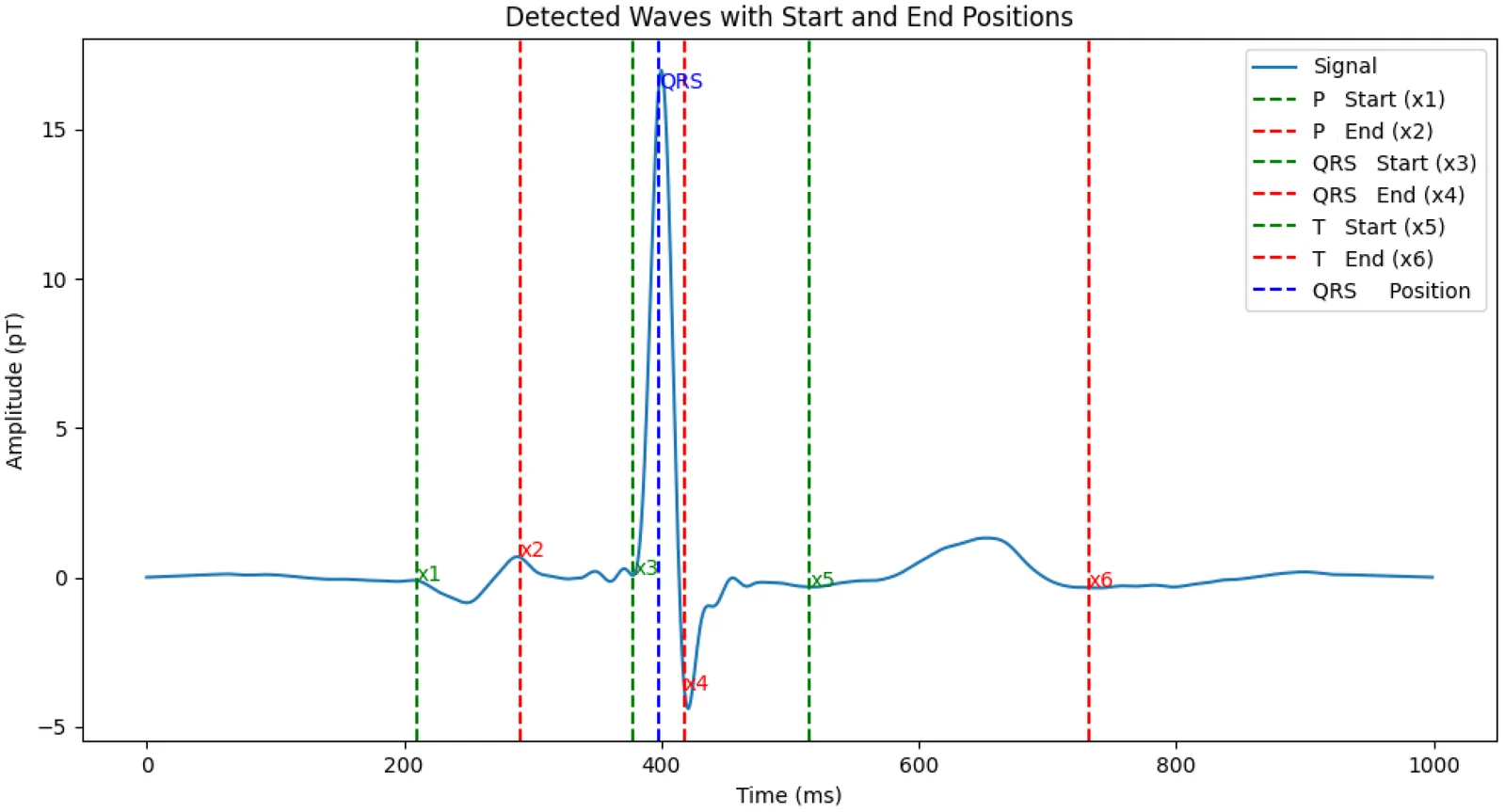
Representative virtual-lead SERF–MCG beat with physiologically defined P, QRS, ST, and T wavebands used for subsequent phase-space reconstruction.


$$x_{n}^{\left(P\right)}[k],\;x_{n}^{\left(QRS\right)}[k],\;x_{n}^{\left(ST\right)}[k],\;x_{n}^{\left(T\right)}[k].$$

The fidelity of the median-beat construction was assessed by comparing the median waveform against the average waveform of consecutive individual heartbeats (*r* = 0.9991) and by demonstrating the reduction of temporal jittering after cross-correlation alignment (detailed results are provided in **Supplementary Figure 1** and **S2**).

### 2.4 Nonlinear phase-space reconstruction and feature extraction

#### 2.4.1 Phase-space reconstruction

To capture nonlinear dynamical structure, each waveband segment was embedded into a high-dimensional phase space using delay-coordinate embedding Takens (1981), as shown in Equation 1:

(1) $$X(i)={\left[x(i),x(i+\tau ),\ldots ,x(i+(m-1)\tau )\right]}^{T},$$

where *m* is the embedding dimension and *τ* is the time delay. In the primary analysis, these parameters were estimated adaptively for each subject-level median beat and then applied consistently to the waveband-resolved phase-space trajectories within that beat, as follows:

• Time delay (*τ*) was selected using an autocorrelation-decay criterion, defined as the first delay at which the autocorrelation decreased below 0.6321 of its zero-lag value.

• Embedding dimension (*m*) was estimated using the False Nearest Neighbors (FNN) method Kennel et al. (1992), with the selected dimension corresponding to the minimum false-neighbor fraction within the tested range.

The adaptive embedding strategy was used as the primary analysis to obtain subject-specific phase-space reconstructions before waveband-resolved feature extraction. To address the mathematical comparability of nonlinear indices across subjects, we additionally performed a fixed-embedding sensitivity analysis using one common coordinate system for all subjects. The cohort-level distribution of the adaptive embedding parameters is reported in **Table 2**.

**Table 2:** Distribution of adaptive phase-space embedding parameters across the cohort.

Parameter set	N	*m* mean ± SD	*m* median [IQR]	*m* mode	*τ* mean ± SD (ms)	*τ* median [IQR] (ms)	*τ* fmode (ms)
Subject-level representative median beat	1848	6.38±1.74	6 [5, 8]	5	12.18±2.54	12 [10, 14]	11

Adaptive embedding parameters were estimated for each subject-level representative median beat and then applied consistently to the waveband-resolved phase-space trajectories within that beat. Values are reported as mean ± SD, median [IQR], and mode.

This yields a distinct attractor $A_{n}^{\left(w\right)}\subset {\mathbb{R}}^{m}$ for each waveband *w*. Representative waveband-specific phase-space trajectories are shown in **Figure 5**.

**Figure 5:**
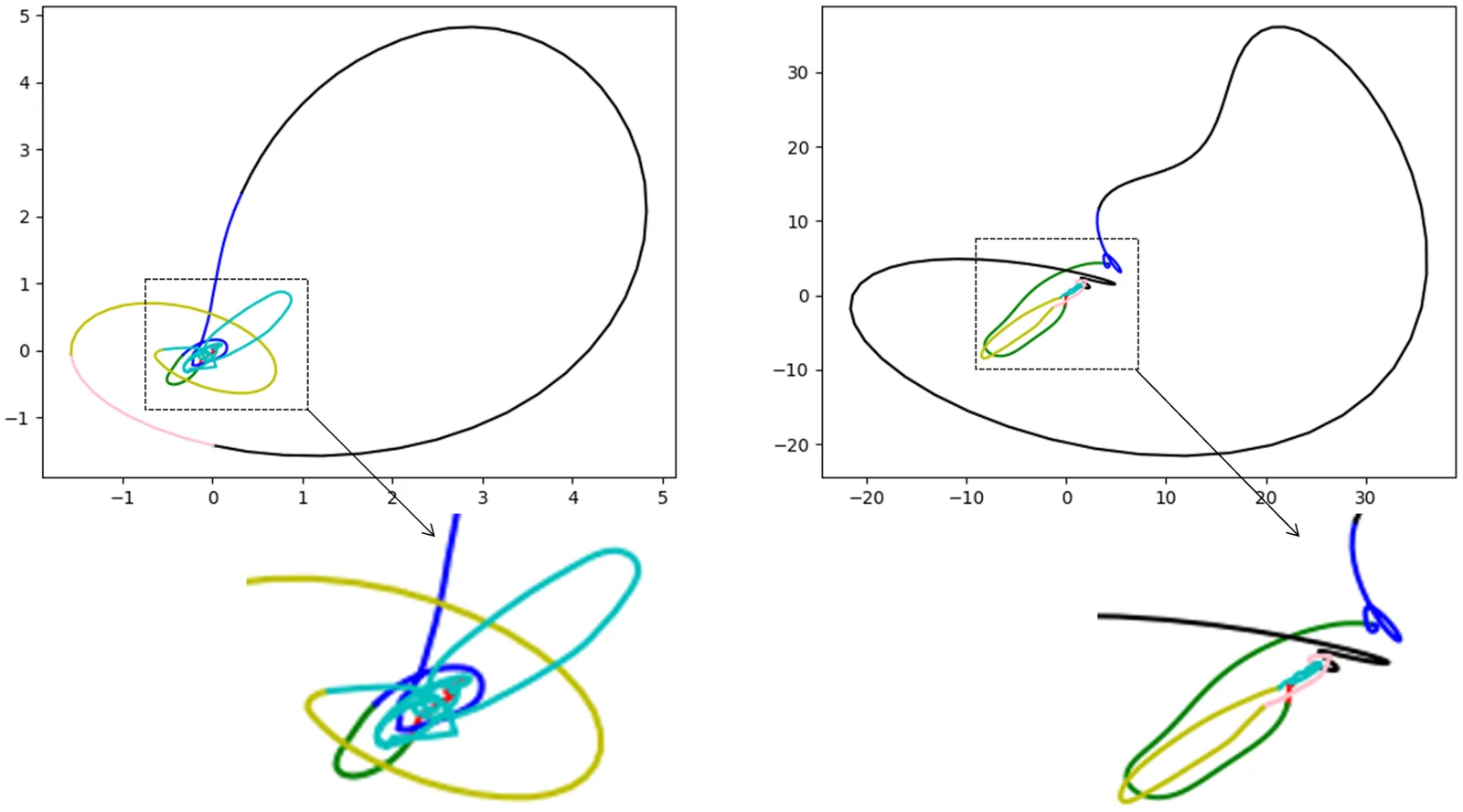
Representative phase-space trajectories (2D projection). The time-domain signals (left) are transformed into phase-space attractors (right) via delay embedding. Note the distinct topological loops corresponding to P, QRS, ST, and T wavebands (ST segment highlighted in pink/yellow), which reveal nonlinear dynamical structures not visible in the linear time series. The zoomed-in inlet explicitly highlights the detailed attractor morphology of the ST-segment and T-wave regions, providing a clearer visualization of the fine-scale dynamical instabilities during repolarization.

#### 2.4.2 Feature extraction

From the reconstructed waveband attractors and the corresponding multi-channel waveband segments, we extracted a total of 32 descriptors per beat, categorized into phase-space geometric descriptors, entropy-related descriptors, Lyapunov-related descriptors, and spatial energy/eigenvalue-ratio descriptors. A summary of all extracted features is provided in **Table 3**.

**Table 3:** Summary of waveband-resolved nonlinear phase-space reconstruction (PSR) features.

Feature category	Wavebands	Descriptors & definitions
**Geometric**	All	**Length** (*L*): Sum of Euclidean distances between consecutive points Equation 2. **Area** (*A*): Enclosed area of 2D projection via shoelace formula. **Shape**: Anisotropy index derived from eigenvalues of the covariance matrix.
**Entropic**	All	**Shannon Entropy** (*H*): Complexity of amplitude distribution. **Sample Entropy** (SampEn): Regularity of trajectory. Because short waveband lengths can reduce estimator reliability, SampEn-based primary interpretation was restricted to the longer repolarizationrelated ST and T wavebands; P and QRS SampEn estimates were not used as key inferential markers.
**Chaotic**	All	**Lyapunov-related descriptor** (*λ*_max_): Sensitivity to initial conditions via the Rosenstein algorithm Equation 4. Because short waveband lengths can reduce estimator reliability, primary interpretation of Lyapunov-related descriptors was focused on the longer ST and T wavebands, while P- and QRS-related estimates were treated as exploratory.
**Energetic**	All	**Energy** (*E*): Sum of squared amplitudes. **Eigenvalue Ratio**: $\lambda_{1}/\sum \lambda_{i}$, quantifying attractor elongation.

A total of 32 descriptors were extracted per beat across the P, QRS, ST, and T wavebands.

##### 2.4.2.1 Geometric descriptors

We computed the trajectory length (*L*), which quantifies the total path excursion of the attractor in phase space:

(2) $$L=\sum_{i=1}^{N-1}\|X_{i+1}-X_{i}\|$$

where **X***_i_* represents the *i*-th point in the embedded phase space and *N* is the number of points. To characterize the attractor’s expansion, we calculated the Enclosed Area (*A*) of its projection onto the first two principal components [(*u,v*)] using the shoelace formula, as shown in Equation 3:

(3) $$A=\frac{1}{2}|\sum_{i=1}^{N-1}(u_{i}v_{i+1}-u_{i+1}v_{i})|.$$

Additionally, the Shape Anisotropy (*S*) was derived from the eigenvalues λ1 ≥ λ2 ≥ ··· ≥ λmof the attractor’s covariance matrix, defined as the ratio of the largest eigenvalue to the total variance $\left(S=\lambda_{1}/\sum_{j=1}^{m}\lambda_{j}\right)$, capturing the tendency of the trajectory to align along a dominant axis.

##### 2.4.2.2 Entropy and chaos

Complexity was quantified using Shannon entropy and Sample Entropy (SampEn) Richman and Moorman (2000); Costa et al. (2002). Sensitivity to initial conditions was measured by the Largest Lyapunov Exponent (*λ*_max_) using the Rosenstein algorithm Rosenstein et al. (1993):

(4) $$\lambda_{\max\;}\approx \frac{1}{\Delta t}\langle \frac{1}{k_{2}-k_{1}}\ln\;\frac{\left\|\delta X(k_{2})\right\|}{\left\|\delta X(k_{1})\right\|}\rangle .$$

In practice, *λ*_max_ was obtained as the slope of the linear region of 〈ln*d*(*k*)〉 versus *k*Δ*t* (Rosenstein method), where *k*_1_ and *k*_2_ index the fitted interval. Because the waveband-specific segments were relatively short, nonlinear descriptors were interpreted with caution. The average segment lengths were approximately 80, 100, 120, and 160 samples for the P, QRS, ST, and T wavebands, respectively. For delay-coordinate embedding with cohort-level median parameters around *m* = 6 and *τ* = 12 ms, the effective number of embedded vectors is reduced relative to the original segment length. Therefore, entropy- and Lyapunov-related descriptors were not interpreted as precise estimates of invariant dynamical quantities, but as standardized signal-derived descriptors computed consistently across subjects. Primary physiological interpretation focused on the longer ST and T wavebands, whereas P- and QRS-related nonlinear estimates were treated as exploratory and were not used as standalone evidence. This limitation is further discussed in the Limitations section.

### 2.5 Analysis of cross-waveband coupling

To investigate whether the clinical patient cohort showed altered signal-level associations among cardiac phases compared with the healthy reference cohort, we employed complementary cross-waveband association measures.

#### 2.5.1 Self-attention encoder

The waveband-specific feature vectors are fed into a self-attention encoder to capture cross-phase dependencies Vaswani et al. (2017). The attention mechanism is defined as shown in Equation 5:

(5) $$\text{Attention}(Q,K,V)=\text{softmax}\;(\frac{QK^{T}}{\sqrt{d_{k}}})V,$$

where Q, K, and V denote the Query, Key, and Value matrices derived from linear projections of the input waveband-level feature matrix H, and $d_{k}$ is the key dimension used for scaling. The matrix $QK^{T}/\sqrt{d_{k}}$ provides attention compatibility scores among cardiac phases, and the row-wise softmax converts these scores into normalized attention weights. In this study, the resulting attention matrix was interpreted as a model-derived association pattern among wavebands, not as direct causal information flow.

The self-attention encoder was trained in a supervised manner using the cohort label only for exploratory group-discrimination analysis. The input to the encoder was a four-node waveband-level feature representation, where each node corresponded to one cardiac phase. Each waveband-specific feature vector was linearly projected to a common hidden dimension of *d_k_*= 32. The encoder used a single scaled dot-product self-attention block over the four waveband nodes, with learnable query, key, and value projections. The attention output was mean-pooled across wavebands and combined with a residual projection before being passed to a classifier head consisting of a 32-to-64 linear layer, ReLU activation, dropout with probability 0.2, and a final 64-to-2 linear layer. The model was optimized using cross-entropy loss with the Adam optimizer, an initial learning rate of 1 × 10^-3^, weight decay of 1 × 10^-4^, batch size of 64, and 30 training epochs.

Because the attention module was trained with group labels, the resulting attention weights are label conditioned model-derived association weights. They should therefore not be interpreted as neutral or intrinsic physiological coupling measures. Instead, they indicate which cross-waveband relationships were emphasized by the supervised model for exploratory group discrimination. For this reason, attention-derived matrices were interpreted together with correlation-based association matrices and were not used as independent evidence of causal physiological coupling.

#### 2.5.2 Correlation and mutual information

To capture statistical dependencies, we computed:

• Pearson Correlation Matrix: $R(w_{1},w_{2})=\text{corr}(x^{\left(w_{1}\right)},x^{\left(w_{2}\right)})$, capturing linear coupling.

• Mutual Information (MI): $I(w_{1};w_{2})$, estimated via a *k*-nearest-neighbor estimator on PCA-compressed representations, capturing nonlinear interdependence Kraskov et al. (2004).

### 2.6 Exploratory classification models and ablation study

To explore the group-discriminative information contained in the proposed features, we trained conventional and ensemble machine-learning models, including logistic regression, SVM with a radial-basis-function kernel, random forest, XGBoost Chen (2016), LightGBM, multilayer perceptron, soft-voting ensemble, and stacking fusion. Classification was used only as an exploratory assessment of signal-level separability between the healthy reference cohort and the heterogeneous clinical patient cohort, not as definitive clinical diagnostic validation.

The dataset was divided into stratified training and held-out test partitions using a 70:30 ratio with a fixed random seed. Numerical features were standardized using parameters estimated from the training partition and then applied to the held-out test partition. For stacking fusion, base learners generated out-of-fold probability estimates on the training partition using five-fold stratified cross-validation. These out-of-fold probabilities were used as meta-features for a logistic-regression meta-learner. Test-set meta-features were obtained by averaging fold-wise base-model predictions, and final performance was evaluated on the held-out test set.

Waveband ablation experiments were performed to assess the relative contribution of phase-specific features. Models were trained on selected feature subsets, including P-only, QRS-only, ST-only, T-only, QRS+ST+T, and all wavebands. These analyses were interpreted as exploratory evidence of signal-level feature contribution rather than as clinical diagnostic validation.

### 2.7 Statistical analysis

Group Comparisons and Evidence. Continuous nonlinear features were summarized as mean ± SD. Group differences between healthy controls and the clinical patient cohort were first assessed using Welch’s *t*-test Welch (1947). Because nonlinear descriptors such as entropy-related indices, Lyapunov-related indices, spatial energy descriptors, and eigenvalue-ratio descriptors may deviate from normality, we additionally assessed distributional normality using the Shapiro–Wilk test and performed Mann–Whitney U tests as non-parametric sensitivity analyses. To complement frequentist *p*-values, we computed Bayes Factors (BF_10_) using a JZS prior. BF_10_ *>* 3 and *>* 10 indicated moderate and strong evidence, respectively.

Multiple Comparison Control. Given the high dimensionality of the feature set, all *p*-values were corrected using the Benjamini–Hochberg False Discovery Rate (FDR) procedure (*q <* 0.05).

Effect Size Estimation. Effect sizes were quantified using Cohen’s *d* for group differences. Standardized univariate logistic regression was used to estimate odds ratios (ORs) per standard deviation increase for each feature. The model is defined as $\log\;[p/(1-p)]=a_{0}+\beta_{1}z$, where *z* is the standardized feature value. These ORs were used only as descriptive univariate association measures and should not be interpreted as independent multivariable predictors because feature collinearity and clinical covariates were not fully modeled.

Feature Collinearity Analysis. To assess interdependence among the extracted nonlinear descriptors, we computed a pairwise Spearman correlation matrix across the extracted feature set. This analysis was used to identify correlated feature groups, particularly among descriptors derived from the same waveband or from related phase-space trajectories. The resulting feature-correlation structure is reported in **Supplementary Figure 3** and interpreted as an exploratory assessment of descriptor redundancy.

AUC Comparison. For exploratory comparison between the morphology-based baseline and the proposed nonlinear feature framework, AUC values were compared using DeLong’s test for correlated ROC curves based on prediction scores from the same held-out test partition. This analysis was used to assess whether the observed AUC difference exceeded what would be expected from sampling variability.

Visualization. To explore the manifold structure of the nonlinear feature space, we employed t-SNE Maaten and Hinton (2008) and Uniform Manifold Approximation and Projection (UMAP). These visualizations were used only for exploratory inspection of feature-space organization and were not used for classifier training, model selection, or diagnostic inference. UMAP was configured with Euclidean distance, *k* = 15 neighbors, and min dist = 0.1.

## 3 Results

### 3.1 Dataset and nonlinear feature characteristics

After strict quality control, 1,848 statistically independent subject-level median SERF–MCG beats were analyzed, including 906 healthy controls and 942 recordings from the heterogeneous clinical cardiovascular patient cohort. We extracted PSR-derived nonlinear descriptors per beat, summarized in **Table 3**.

Group differences were non-uniform across the cardiac cycle. While P-wave descriptors showed relatively small differences, repolarization-related wavebands, particularly ST and T, showed statistically significant but partially overlapping group differences. Specifically, recordings from the clinical patient cohort showed lower ST sample entropy, altered Lyapunov-related descriptors, and altered energy-related descriptors in the ST/T segments (**Table 4**). These results describe cohort-level signal-feature differences and should not be interpreted as ischemia-specific causal effects.

**Table 4:** Group-wise statistics of key PSR features.

Feature	Control	Clinical patient	Cohen’s *d*	*p_t_*	OR per SD
P entropy	3.87 ± 0.45	3.81 ± 0.46	−0.14	1.9×10^-3^	0.87
QRS len	25.23 ± 11.38	22.28 ± 11.53	−0.26	3.6×10^-8^	0.77
QRS shape	2.29 ± 1.80	2.89 ± 2.43	0.28	3.0×10^-9^	1.37
ST len	3.84 ± 2.90	4.47 ± 3.29	0.20	1.4×10^-5^	1.23
ST sampen	0.105 ± 0.080	0.085 ± 0.068	−0.27	1.2×10^-8^	0.76
ST lyap	0.023 ± 0.029	0.027 ± 0.029	0.14	6.8×10^-4^	1.17
ST energy	3.54 ± 4.73^†^	4.16 ± 5.97^†^	0.12	1.4×10^-2^	1.12
T len	4.73 ± 3.03	4.06 ± 3.00	−0.22	2.5×10^-6^	0.80
T entropy	4.06 ± 0.40	4.01 ± 0.43	−0.12	9.0×10^-3^	0.89
T energy	6.42 ± 7.21^†^	4.14 ± 5.92^†^	−0.35	1.8×10^-13^	0.69

† Energy values scaled by 10^4^ for display.

Values are mean ± SD. p_t_: Welch’s t-test p-value; OR: standardized univariate odds ratio per SD increase. Non-parametric sensitivity results are provided in **Supplementary Table 1**. The clinical patient cohort showed lower ST sample entropy and altered Lyapunov-related descriptors in the ST segment; however, the modest Cohen’s d values indicate partially overlapping feature distributions.

Feature-level statistics are visualized in the forest plot (**Figure 6**). ST sample entropy and T-wave energy survived Benjamini–Hochberg FDR correction (*q <* 0.05), although the corresponding Cohen’s *d* values indicated small-to-moderate standalone effect sizes and substantial overlap between groups. Therefore, these features were interpreted as exploratory cohort-level signal descriptors rather than standalone clinical discriminators.

**Figure 6:**
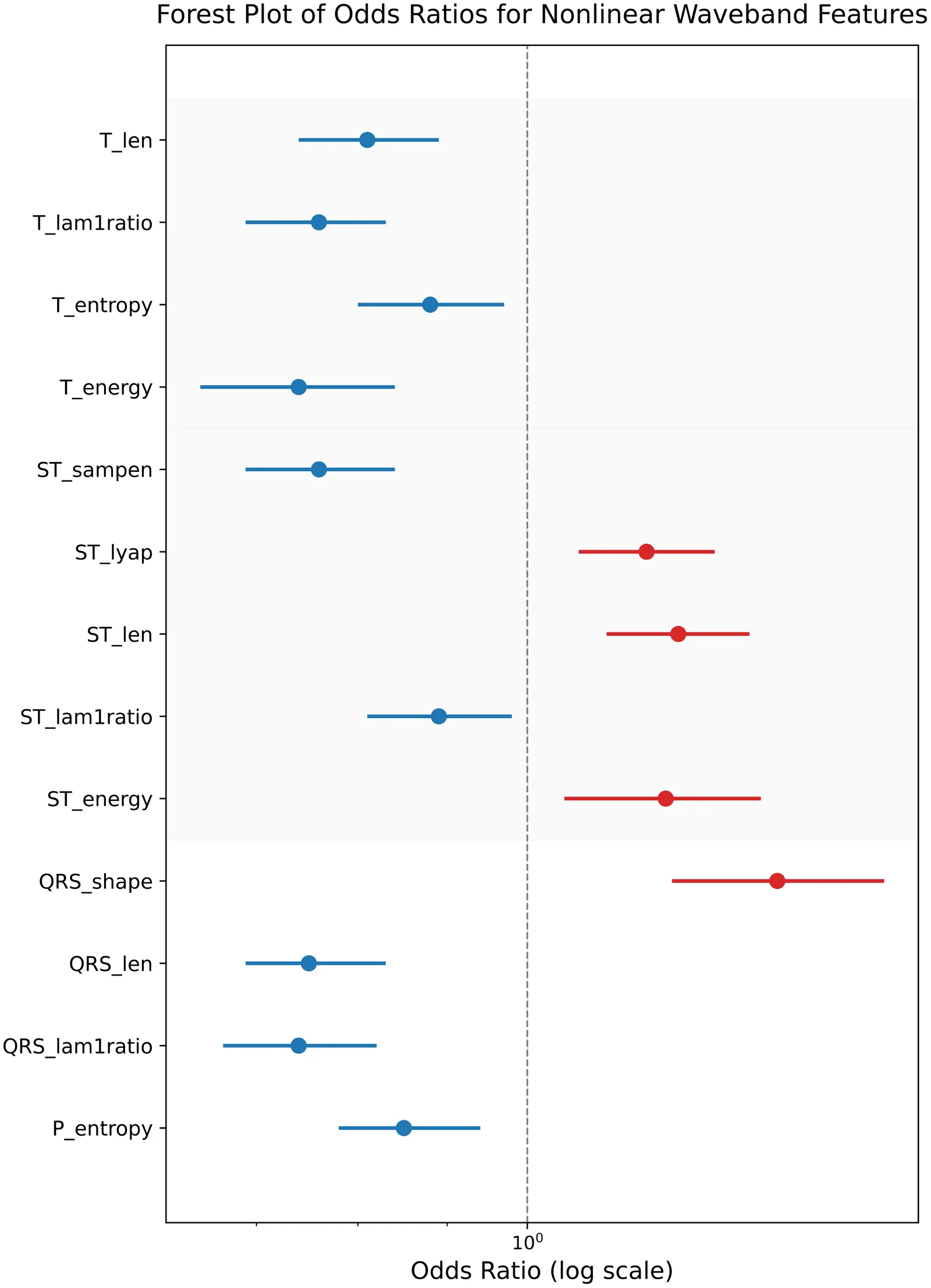
Forest plot of standardized univariate odds ratios. Features with OR *>* 1 indicate higher odds of belonging to the clinical patient cohort per standard deviation increase. The odds ratios are descriptive univariate association measures and should not be interpreted as independent multivariable predictors because feature collinearity and clinical covariates were not fully modeled.

Because Shapiro–Wilk testing indicated deviations from normality across the extracted feature set in both cohorts, Mann–Whitney U tests were performed as non-parametric sensitivity analyses. The main repolarization-related findings remained directionally consistent under non-parametric testing. Multiple features remained significant after Benjamini–Hochberg FDR correction using the Mann–Whitney U test, including ST sample entropy, ST Lyapunov-related descriptor, T-wave energy, T-wave length, and T-wave entropy. The full sensitivity analysis, including Shapiro–Wilk tests, Welch’s *t*-tests, Mann–Whitney U tests, and FDR-adjusted *p*-values, is provided in **Supplementary Table 1**.

To address the concern regarding the mathematical comparability of entropy- and Lyapunov-related descriptors, we quantified the adaptive embedding-parameter distribution across all 1,848 subjects (**Table 2**). The adaptive embedding dimension was *m* = 6.38 ± 1.74 with a median of 6 [5, 8] and a mode of 5, 357 while the adaptive delay was *τ* = 12.18 ± 2.54 ms with a median of 12 [10, 14] ms and a mode of 11 ms. We additionally conducted a fixed-embedding sensitivity analysis using a common coordinate system for all subjects. Under this standardized setting, the main ST/T-related group differences were directionally preserved. These results suggest that the observed repolarization-related signal differences were not solely driven by subject-specific variation in embedding parameters.

### 3.2 Group-level differences in cross-waveband feature associations

We next examined whether the clinical patient cohort showed altered signal-level associations among cardiac phases compared with the healthy reference cohort. To this end, cross-waveband feature associations were evaluated from two complementary perspectives, as illustrated in **Figure 7**.

**Figure 7:**
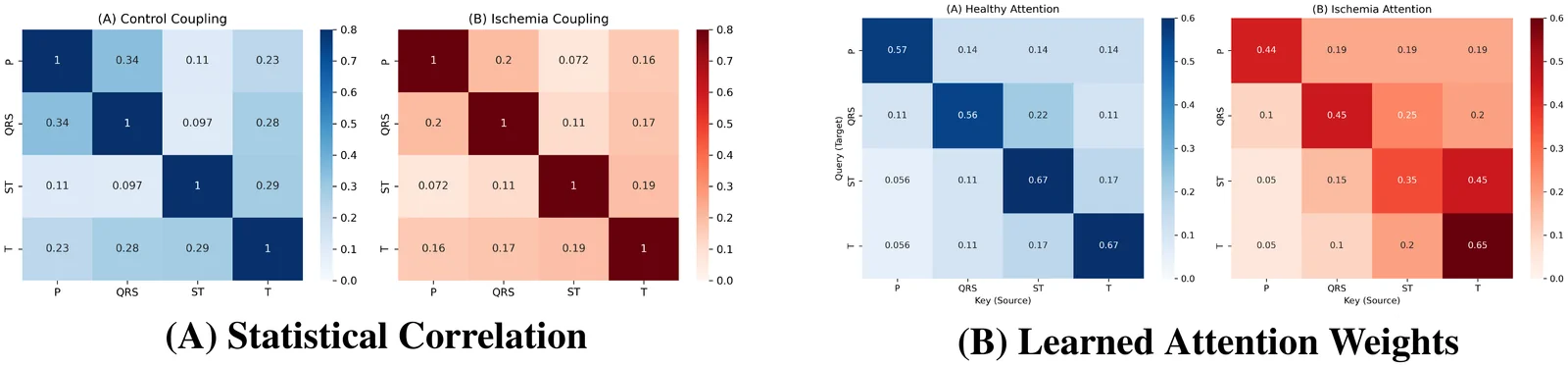
Dual-perspective visualization of cross-waveband feature associations. **(A)** Correlation matrices derived from standardized waveband-level feature aggregates for the healthy reference cohort and the heterogeneous clinical patient cohort, as explicitly labeled within the heatmaps. **(B)** Mean attention matrices derived from the supervised four-node waveband self-attention encoder. Because the attention model was trained with group labels, these weights represent label-conditioned model-derived association patterns rather than neutral physiological coupling or causal information flow. Together, the correlationbased and attention-derived views provide exploratory evidence that repolarization-related ST/T feature relationships contributed to group-level signal separability in this retrospective cohort.

First, the statistical correlation analysis (**Figure 7A**) showed a higher ST–T association pattern in the clinical patient cohort than in the healthy reference cohort, based on the group labels shown within the heatmaps.

Second, the supervised attention-derived weights (**Figure 7B**) showed a consistent ST–T association pattern emphasized by the model. Because the attention encoder was trained with group labels, these weights should be interpreted as label-conditioned model-derived association patterns rather than as direct physiological coupling or causal information flow.

The convergence between the correlation-based and attention-derived analyses suggests that ST/T feature relationships contributed to group-level signal separability in this retrospective cohort. These findings remain exploratory and do not establish an ischemia-specific causal mechanism.

### 3.3 Nonlinear manifold structure

To explore the global structure of the high-dimensional feature space, we employed t-SNE and UMAP for dimensionality reduction. As shown in **Figure 8**, the t-SNE embedding showed a partial separation tendency between the healthy reference cohort and the clinical patient cohort, suggesting that the extracted nonlinear descriptors contain group-discriminative signal information. This visualization was used only for exploratory inspection and was not used for model training, model selection, or diagnostic inference.

**Figure 8:**
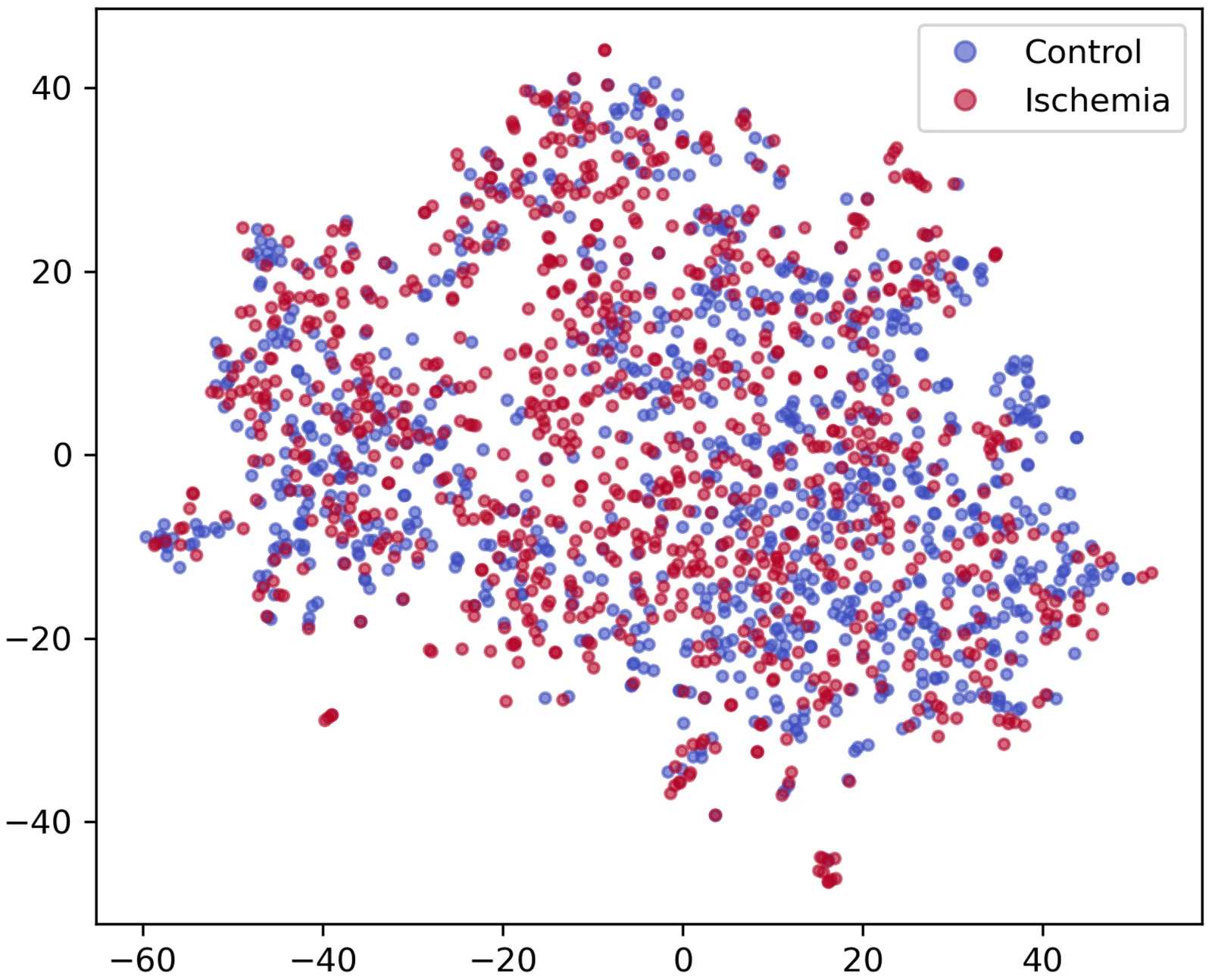
t-SNE embedding of the nonlinear feature space. Each point represents one subject-level representative SERF–MCG beat. The visualization shows a partial separation tendency between the healthy reference cohort and the heterogeneous clinical patient cohort, suggesting that the extracted wavebandspecific nonlinear features contain group-discriminative signal information. This visualization is intended for exploratory interpretation and was not used as a standalone diagnostic criterion.

Further visualization was performed using UMAP (**Figure 9**). **Figure 9A** shows the feature manifold colored by cohort label, whereas **Figure 9B** shows the same manifold colored by ST sample entropy.

**Figure 9:**
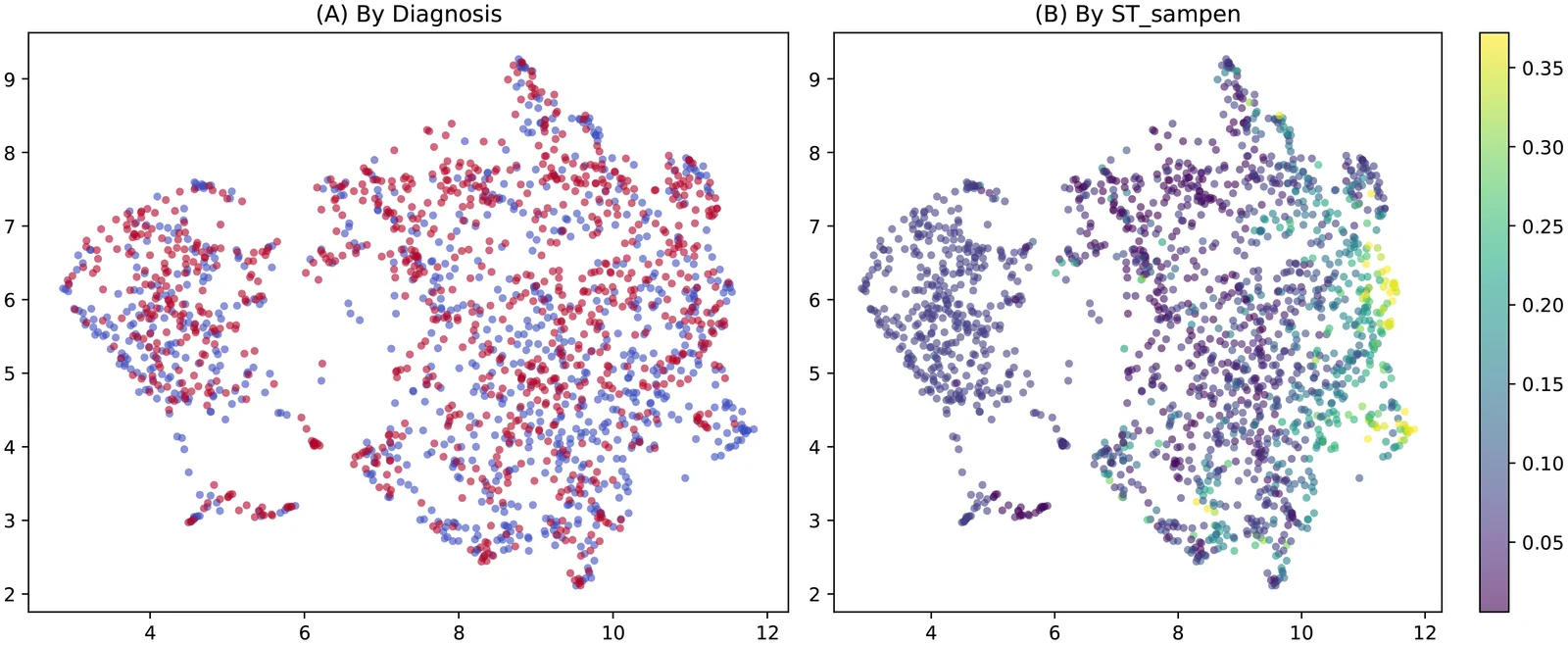
Physiologically interpretable UMAP visualization. **(A)** The feature manifold colored by cohort label shows a partial clustering tendency of clinical patient recordings. **(B)** The same manifold colored by ST sample entropy values. Regions enriched for clinical patient recordings tended to correspond to lower ST sample entropy values. This exploratory visualization is consistent with group-level differences in repolarization-related signal complexity but does not establish ischemia-specific diagnostic or causal evidence.

Regions enriched for clinical patient recordings tended to overlap with regions showing lower ST sample entropy. This visual pattern is consistent with the statistical observation of lower repolarization-related complexity in the clinical patient cohort, but it should be interpreted as exploratory rather than confirmatory evidence.

### 3.4 Discriminative power of waveband components

Although clinical diagnosis was not the primary aim, a waveband ablation study (**Table 5**) was conducted to quantify the exploratory group-discriminative information contained in each phase and to provide a benchmark comparison with morphology-based MCG descriptors.

**Table 5:** Waveband ablation and baseline comparison study. Performance metrics for classifiers trained on different subsets of the cardiac cycle.

Feature Subset	ACC	AUC	Sens	Spec	F_1_
Morphological Baseline	0.571	0.614	0.587	0.555	0.582
Full nonlinear framework	0.699	0.754	0.721	0.676	0.710
P only	0.58	0.601	0.56	0.60	0.57
QRS only	0.58	0.602	0.57	0.59	0.58
ST only	0.59	0.628	0.59	0.60	0.60
T only	0.61	0.648	0.62	0.61	0.62
QRS + ST + T	**0.66**	**0.708**	**0.67**	**0.65**	**0.67**
All wavebands	0.67	0.720	0.68	0.66	0.68

The morphology-only baseline uses standard amplitude-based MCG descriptors. The full nonlinear framework achieved a higher held-out test AUC than the morphologyonly baseline, and this difference was formally assessed using DeLong’s test. These results quantify signal-level separability and should not be interpreted as clinical diagnostic validation.

- Comparison with a morphology-based baseline: To provide a simple benchmark, we trained a baseline model using standard MCG morphological descriptors, including ST-segment deviation and T-wave amplitude-related measures extracted from the same median beats. The proposed nonlinear framework showed a higher held-out test AUC than the morphology-only baseline (0.754 vs. 0.614). A formal DeLong comparison using prediction scores from the same held-out test partition confirmed that this AUC difference was statistically significant (*z* = 5.477, *p* = 4.33 × 10^-8^).
- Repolarization-related contribution: Models using only ST or T features showed higher AUCs than models using P-wave features, suggesting that repolarization-related descriptors contributed more to group discrimination in this dataset.
- Combined waveband information: Combining QRS, ST, and T wavebands yielded the highest PSR-only performance (AUC ≈ 0.71), suggesting that multi-phase information provided complementary discriminative value.
- Attention-related gain: The inclusion of self-attention-derived association features provided a modest improvement over baseline PSR features (**Figure 10**). This finding suggests that cross-waveband association patterns may contain additional information, although the improvement should be interpreted cautiously given the moderate absolute AUC.

**Figure 10:**
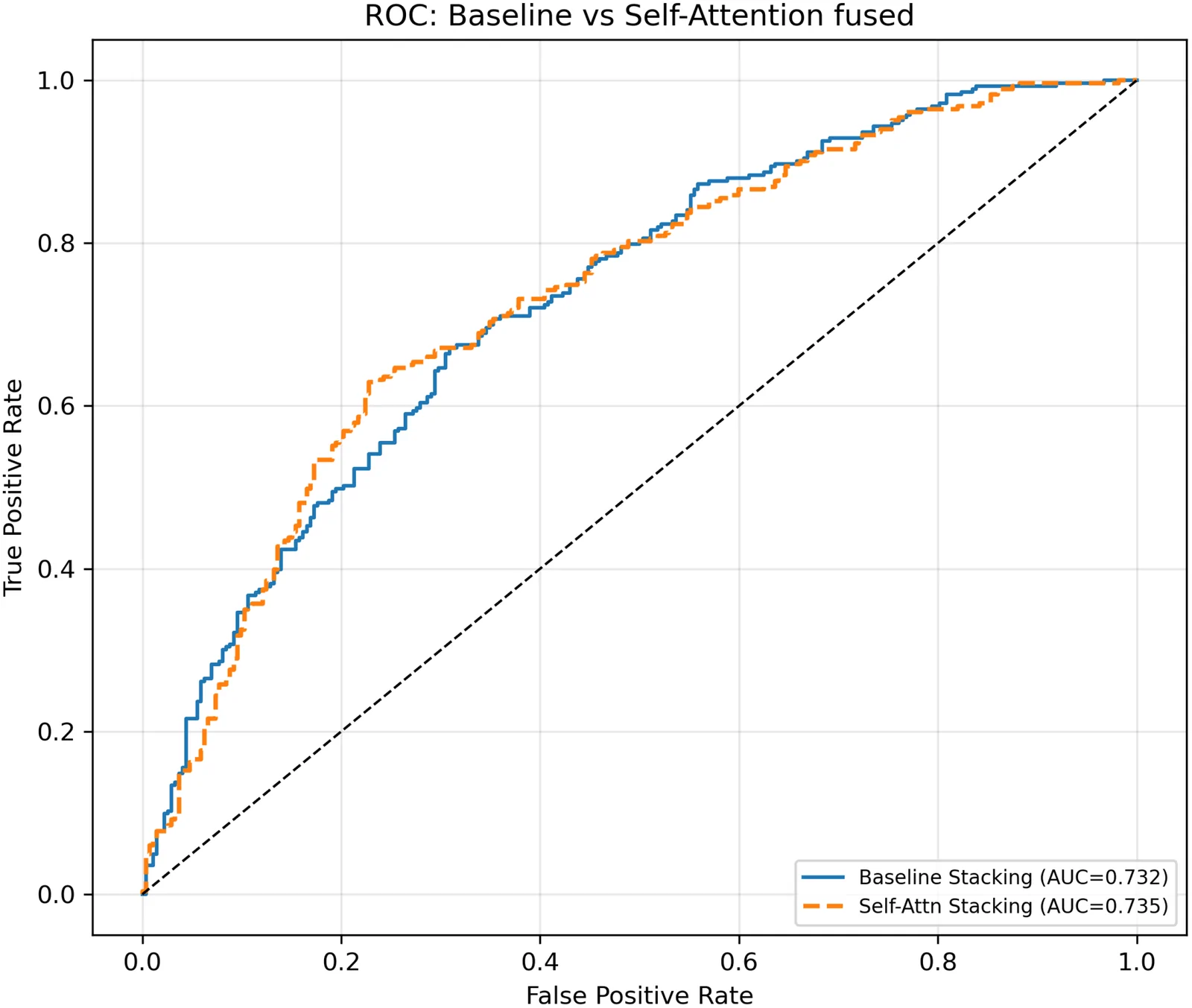
ROC curves for exploratory feature comparison. The proposed nonlinear framework achieved a higher held-out test AUC than the morphology-based baseline (0.754 vs. 0.614). DeLong’s test for correlated ROC curves using prediction scores from the same held-out test partition indicated that the AUC difference was statistically significant (*z* = 5.477, *p* = 4.33 × 10^-8^). Because the absolute AUC remains moderate and the cohort was retrospective and heterogeneous, this comparison is interpreted as exploratory evidence of additional group-discriminative signal information rather than definitive clinical diagnostic validation.

## 4 Discussion

This study analyzes SERF–MCG beats through the lens of waveband-resolved nonlinear dynamics, aiming to determine whether phase-space features can characterize signal-level differences between stringently screened healthy adults and a heterogeneous clinical cardiovascular patient cohort. By decomposing the cardiac cycle into P, QRS, ST, and T phase-space trajectories, we observed group-level differences in repolarization-related nonlinear descriptors and ST–T feature associations. Because the dataset was retrospective, de-identified, and clinically heterogeneous, these findings should be interpreted as exploratory signal-level observations rather than ischemia-specific causal or diagnostic evidence.

### 4.1 Interpretation of repolarization-related signal differences

The observed feature pattern suggests that recordings from the clinical patient cohort were associated with lower repolarization-related complexity and altered local instability-related descriptors in the ST and T wavebands. This pattern is broadly consistent with the “loss of complexity” hypothesis in pathological physiology Goldberger et al. (2002), although the present retrospective signal-level analysis cannot establish a direct cellular mechanism or ischemia-specific origin.

From a physiological perspective, ischemia and other cardiovascular stressors may impair metabolic reserves, alter ion-channel function, and constrain repolarization dynamics. The reduced ST sample entropy may therefore reflect lower signal complexity, whereas altered Lyapunov-related descriptors may indicate changes in local trajectory divergence. These interpretations are hypothesis-generating and require validation against complete clinical, imaging, and electrophysiological data.

### 4.2 Cross-waveband association as an exploratory signal marker

A key observation of this work is the altered association between the ST and T wavebands in the clinical patient cohort. Both correlation analysis and the supervised self-attention model showed ST–T association patterns that contributed to group-level signal separability.

Physiologically, this pattern is compatible with the concept that repolarization-related phases may become more constrained under cardiovascular stress. However, because the attention model was supervised by cohort labels and the clinical patient cohort was heterogeneous, the observed ST–T association should be interpreted as an exploratory signal-level marker rather than as direct proof of ischemia-specific repolarization coupling or a causal electrophysiological pathway.

### 4.3 Interpretability vs. black-box learning

In this study, the self-attention mechanism was used as a structured representation-learning tool to summarize associations among waveband-level features. Unlike fully end-to-end black-box models, the four-node architecture constrains the learned representation to predefined cardiac phases, allowing the resulting attention matrix to be inspected in relation to physiologically meaningful wavebands. However, because the attention encoder was trained using cohort labels, attention weights are label-conditioned model-derived association weights. They should not be interpreted as neutral physiological coupling, causal information flow, or direct evidence of electrophysiological interaction. Rather, they provide supervised association patterns that complement the correlation-based analysis and help identify which cross-waveband relationships contributed to exploratory group discrimination.

### 4.4 Limitations and future directions

Several limitations should be acknowledged.

First, the retrospective and de-identified nature of the database limited the available clinical covariates. Exact individual-level demographic variables, including age, sex, BMI, medication status, and detailed comorbidity profiles, were irreversibly unlinked from the raw SERF–MCG waveform files during multi- year data aggregation. Consequently, we could not construct a conventional baseline characteristics table with group-wise means and standard deviations, nor could we perform propensity-score matching or multivariable adjustment for demographic, medication-related, or comorbidity-related confounders. To improve transparency, we provided a cohort-level clinical-characterization and de-identification table (**Table 1**). In addition, the healthy control cohort was defined using stringent inclusion and exclusion criteria to reduce the likelihood of overt cardiovascular disease, major cardiovascular risk factors, prescription-medication effects, or abnormal ECG/echocardiographic findings in the reference group. Nevertheless, residual confounding cannot be excluded. Future prospective studies should collect complete demographic, medication, laboratory, and imaging data to enable matched analyses and covariate-adjusted validation of the proposed nonlinear SERF–MCG biomarkers.

Relatedly, the asymmetric cohort definition introduces a risk of spectrum bias. The healthy control group was stringently screened to exclude cardiovascular disease, major risk factors, prescription medication use, and abnormal ECG or echocardiographic findings, whereas the clinical patient cohort consisted of heterogeneous real-world cardiovascular patients enriched for clinically diagnosed ischemia. Therefore, the observed classification performance may overestimate performance in a clinically realistic screening setting where controls include patients with non-ischemic cardiovascular symptoms or comorbidities. This limitation was emphasized in the revised framing, and the classification results were interpreted only as exploratory signal-level separability rather than diagnostic validation.

In addition, the exact diagnostic modality and timing relative to ischemic symptoms could not be reconstructed for each individual waveform after de-identification. The clinical patient cohort should therefore be interpreted as a heterogeneous cardiovascular cohort enriched for clinically diagnosed ischemia rather than as a uniformly angiography-confirmed ischemia cohort. Potential label heterogeneity and label noise may attenuate, distort, or confound the observed signal differences. Prospective studies with uniform diagnostic verification are required before ischemia-specific conclusions can be drawn.

Second, regarding the effect size, while the Cohen’s *d* values for individual nonlinear features are moderate, these dynamical markers are not intended to replace traditional clinical tools such as troponin testing, ECG interpretation, or morphology-based MCG analysis. Instead, they may provide complementary physiological information. Whether combining these dynamical indices with established clinical risk scores can improve diagnostic performance requires prospective validation.

Third, we used a spatially averaged virtual lead constructed from all 36 SERF–MCG channels. This strategy was chosen to improve global signal stability and cross-subject comparability for the present phase-space analysis. However, spatial averaging may also attenuate localized magnetic-field patterns and high-spatial-frequency information that could be relevant to ischemic territory localization. Therefore, the present study should be interpreted as a global virtual-lead analysis rather than a full spatiotemporal MCG mapping study. Future work should extend the framework to channel-resolved and multi-channel spatiotemporal embeddings.

Fourth, although the use of a magnetically shielded room, band-pass filtering, full-channel averaging, template-based beat rejection, and median-beat construction was intended to improve signal stability, we did not perform a systematic simulated SNR-degradation experiment to determine the exact noise threshold at which phase-space geometry or entropy estimates become unreliable. Because entropy-related descriptors, Lyapunov-related descriptors, and phase-space energy/eigenvalue-ratio indices can be sensitive to noise level and sampling conditions, future work should include controlled noise-injection experiments and SNR-stratified validation to define reliability boundaries for SERF–MCG nonlinear descriptors.

Furthermore, because the waveband-specific segments were short, especially for the P and QRS wavebands, entropy- and Lyapunov-related descriptors were treated as standardized signal-derived descriptors rather than precise estimates of invariant dynamical quantities. Primary interpretation was therefore focused on the relatively longer ST and T wavebands, and short-segment estimates were interpreted cautiously.

Fifth, our analysis focused on a representative median beat for each subject. This design reduced transient noise and avoided within-subject pseudo-replication, but it also suppressed beat-to-beat variability, including micro-instabilities and temporal jitter that may themselves carry nonlinear information. Although waveform-level consistency between the median beat and the average beat was assessed, future studies should directly compare nonlinear features derived from median beats with features averaged across individual beats.

### 4.5 Potential for clinical translation and performance enhancement

As observed in **Table 4**, individual nonlinear indices showed statistically significant group-wise differences but small-to-moderate effect sizes and partially overlapping distributions. Therefore, these descriptors should not be interpreted as standalone diagnostic criteria. Instead, they may provide complementary signal-level information that could be integrated with conventional clinical markers, ECG findings, and morphology-based MCG descriptors in future prospective studies.

The exploratory comparison with a morphology-only baseline (**Table 5**) suggests that nonlinear waveband-resolved features may add group-discriminative signal information beyond simple amplitude-based descriptors. This difference was formally supported by DeLong’s test on the held-out test partition. However, the absolute AUC remained moderate, the cohort was retrospective and heterogeneous, and no external validation cohort was available. Future studies should evaluate whether combining nonlinear SERF–MCG features with complete clinical covariates, laboratory markers, and imaging findings can improve risk stratification in a prospectively collected cohort.

## 5 Conclusions

This study presents a waveband-resolved nonlinear analysis framework for SERF–MCG signal characterization. In this retrospective and de-identified dataset, the framework identified exploratory group-level differences in repolarization-related nonlinear descriptors and cross-waveband association patterns between stringently screened healthy controls and a heterogeneous clinical cardiovascular patient cohort enriched for clinically diagnosed ischemia. These findings support the methodological feasibility of phase-space reconstruction and supervised attention-assisted association analysis for SERF–MCG signal interpretation. However, because individual-level clinical covariates, uniform diagnostic verification, and clinically matched controls were unavailable, the present findings should not be interpreted as ischemia-specific causal evidence or definitive diagnostic validation. Prospective studies with complete demographic, clinical, medication, laboratory, and imaging data are required to validate the clinical utility and generalizability of these nonlinear SERF–MCG markers.

## Data Availability

The datasets presented in this article are not readily available because The MCG data of the subjects used in this study cannot be shared due to privacy. Requests to access the datasets should be directed to Min Xiang, xiang_min@buaa.edu.cn.

## Supplementary material

Supplementary Figure 1 Median-beat construction and beat-alignment validation. These figures show the consistency between the representative median beat and the average waveform of consecutive individual heartbeats, as well as the reduction of temporal jitter after cross-correlation alignment.

Supplementary Figure 2 Median-beat construction and beat-alignment validation. These figures show the consistency between the representative median beat and the average waveform of consecutive individual heartbeats, as well as the reduction of temporal jitter after cross-correlation alignment.

Supplementary Table 1 Non-parametric sensitivity analysis of waveband-resolved nonlinear features. The table reports Shapiro–Wilk normality tests, Welch’s *t*-tests, Mann–Whitney U tests, and FDR-adjusted *p*-values for each feature.

Supplementary Figure 3 Spearman correlation matrix of the extracted waveband-resolved features. The matrix illustrates feature collinearity and descriptor redundancy across wavebands and feature categories.
