# Supplementary material for: Waveband-resolved nonlinear characterization of SERF-magnetocardiography signals: a phase-space reconstruction framework for exploratory clinical cohort analysis
Source: Front Physiol. 2026 Sep 17;17:1770392. doi: 10.3389/fphys.2026.1770392 (PMC13627006; doi:10.3389/fphys.2026.1770392)
Supplement: Supplementary file 1 [file Supplementaryfile1.pdf]

Supplementary Material

Waveband-Resolved Nonlinear Characterization of SERF-Magnetocardiography Signals: A Phase-Space Reconstruction Framework for Exploratory Clinical Cohort Analysis

Keyi Li [1,2], Xiangyang Zhou [1,3], Jiaojiao Pang [6,7,8,9], Rui Shang [6,7,8,9], Yadan Zhang [5], Yangyang Cui [5], Dong Xu [5]*, Min Xiang [1,3,4]*

[1] Key Laboratory of Ultra-Weak Magnetic Field Measurement Technology, Ministry of Education, School of Instrumentation and Optoelectronic Engineering, Beihang University, Beijing, China
[2] Zhejiang Provincial Key Laboratory of Ultra-Weak Magnetic-Field Space and Applied Technology, Hangzhou Innovation Institute, Beihang University, Hangzhou, China
[3] Hefei National Laboratory, Hefei, China
[4] State Key Laboratory of Traditional Chinese Medicine Syndrome / National Institute of Extremely-Weak Magnetic Field Infrastructure, Hangzhou, China
[5] Hangzhou Institute of National Extremely-Weak Magnetic Field Infrastructure, Hangzhou, China
[6] Department of Emergency Medicine, Qilu Hospital of Shandong University, Jinan, China
[7] Shandong Key Laboratory for Magnetic Field-free Medicine & Functional Imaging, National Medicine-Engineering Interdisciplinary Industry-Education Integration Innovation Platform, Qilu Hospital of Shandong University, 250012, Jinan, China
[8] Shandong Provincial Clinical Research Center for Emergency and Critical Care Medicine, Institute of Emergency and Critical Care Medicine of Shandong University, Chest Pain Center, Qilu Hospital of Shandong University, 250012, Jinan, China
[9] National Medical Products Administration (NMPA) Key Laboratory for Clinical Research and Evaluation of Innovative Drug, Qilu Hospital of Shandong University, 250012, Jinan, China

* Corresponding authors: Dong Xu; Min Xiang
Emails: mrwoodeast@126.com; xiang_min@buaa.edu.cn

S1 Validation of Cross-Correlation Alignment

To reduce temporal jitter during R-peak alignment and to limit potential artificial waveform smearing during median-beat construction, we applied cross-correlation maximization before computing the representative median beat.

Supplementary Figure S1 illustrates the effect of this alignment procedure. Raw segmented beats with natural temporal jittering (left) can produce a visibly smeared median waveform. After cross-correlation alignment (right), the retained heartbeats show improved phase consistency, and the resulting median beat better preserves the dominant waveform morphology. This analysis supports the use of alignment as a jitter-reduction step, although it does not eliminate all possible residual phase variability.

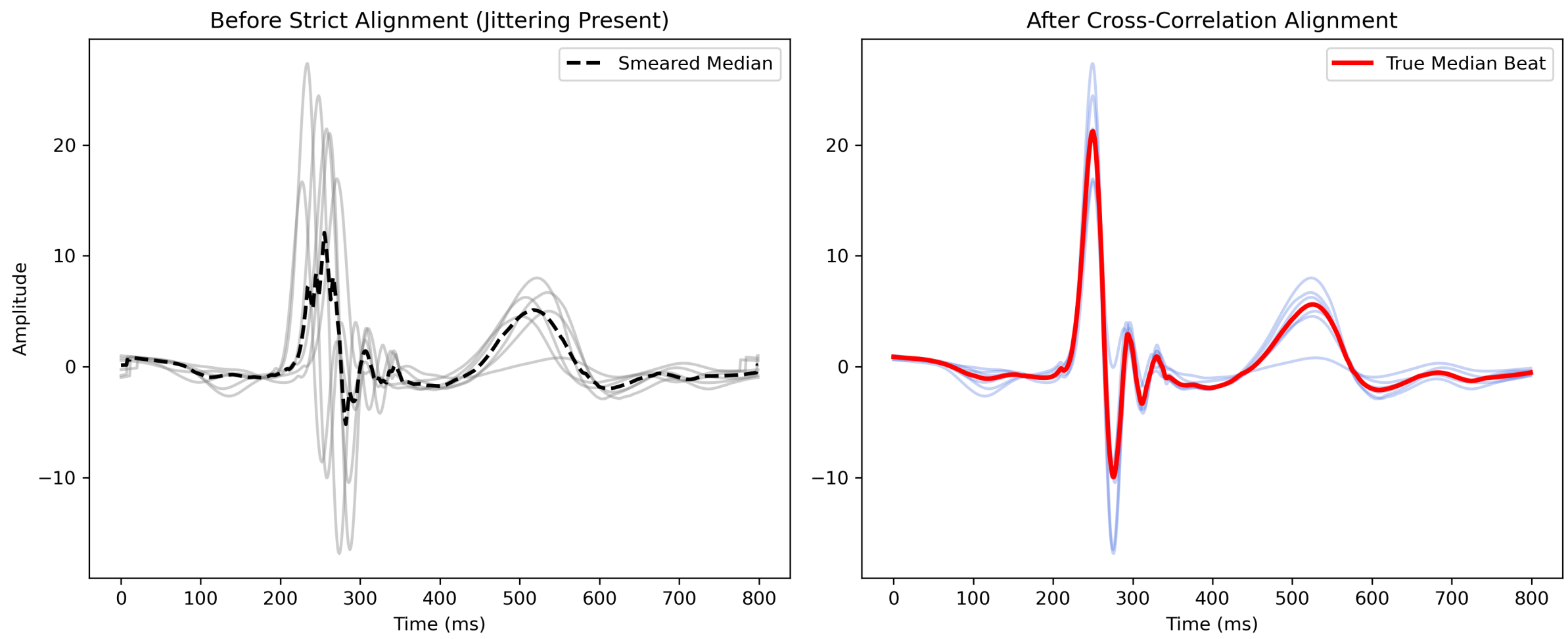

Supplementary Figure S1. Superimposed individual cardiac cycles before (left) and after (right) cross-correlation alignment. The red solid line represents the resulting median beat used for phase-space reconstruction.

S2 Waveform-Level Consistency of the Median Beat

To assess waveform-level consistency of the representative median beat, we compared the point-by-point amplitude of the median waveform with the mean waveform computed from all aligned valid single heartbeats for a representative subject.

As shown in **Supplementary Figure S2**, the median waveform was highly correlated with the mean waveform of aligned valid beats ($r = 0.9991$). This result supports waveform-level consistency between the median and average beat representations. However, this analysis should not be interpreted as feature-level equivalence between nonlinear features extracted from median beats and features averaged across individual beats. Such feature-level validation should be performed in future studies.

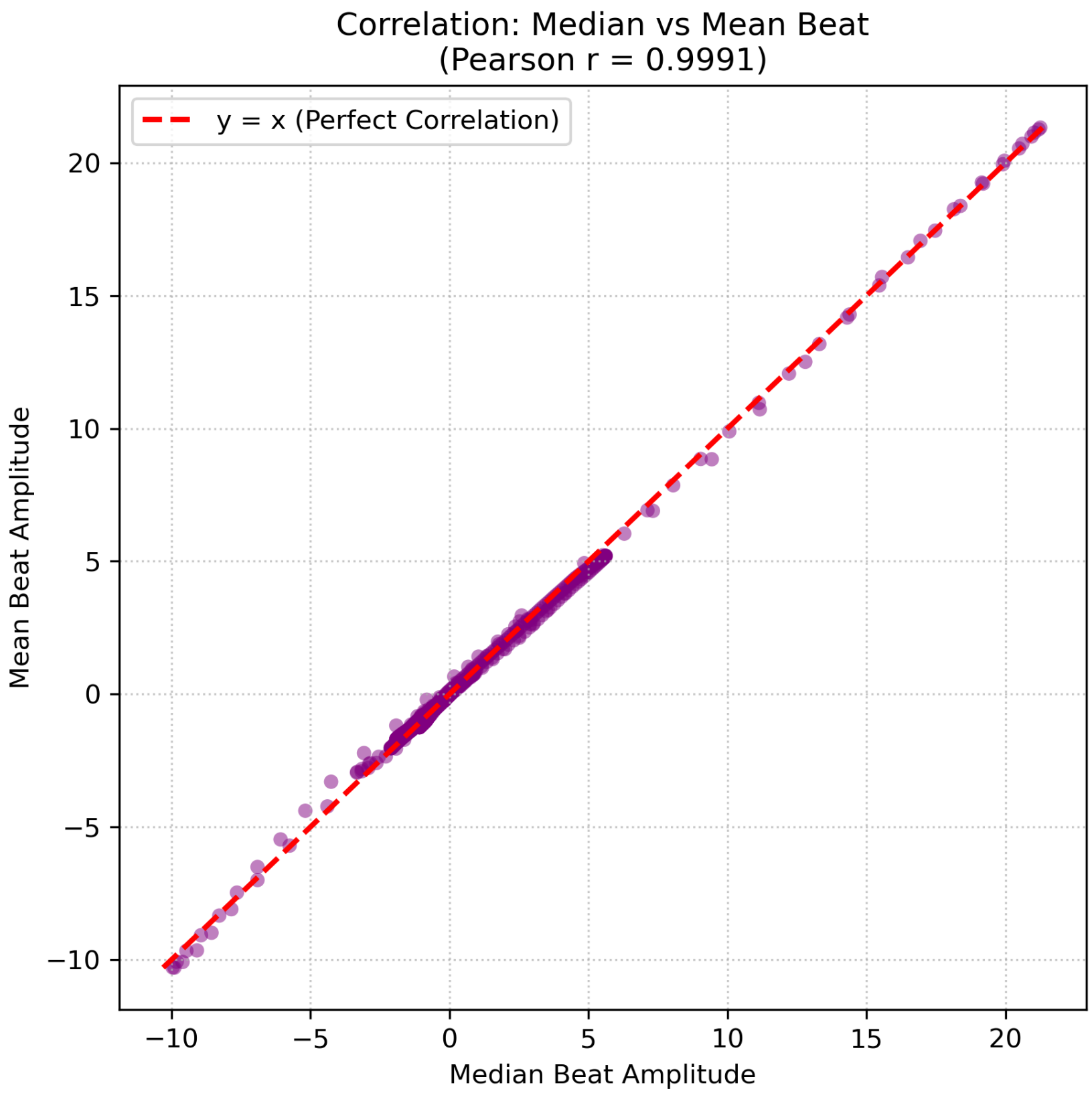

Supplementary Figure S2. Scatter plot showing the point-by-point amplitude correlation ($r = 0.9991$) between the representative median beat and the mean waveform of all aligned valid single heartbeats for a representative subject. The red dashed line represents the identity line ($y = x$).

Supplementary Table S1. Non-parametric sensitivity analysis of representative waveband-resolved nonlinear features. Values are summarized as mean ± SD. Shapiro–Wilk tests were used to assess normality within each cohort, and Mann–Whitney U tests were used as non-parametric sensitivity analyses. FDR adjustment was performed using the Benjamini–Hochberg procedure. The full numerical table is provided as a supplementary CSV file. Energy values are scaled by 10^4 for display where indicated.

**Feature**	**Control**	**Clinical patient**	**Cohen's d**	**MW p**	**MW FDR**
P_entropy	3.87 ± 0.45	3.81 ± 0.46	-0.145	1.50×10^{-3}	3.10×10^{-3}
QRS_len	25.2 ± 11.4	22.3 ± 11.5	-0.257	2.36×10^{-9}	1.26×10^{-8}
QRS_shape	2.29 ± 1.80	2.89 ± 2.43	0.276	1.73×10^{-14}	2.77×10^{-13}
ST_len	3.84 ± 2.90	4.47 ± 3.29	0.202	1.07×10^{-5}	2.63×10^{-5}
ST_sampen	0.105 ± 0.080	0.085 ± 0.068	-0.270	2.68×10^{-11}	2.14×10^{-10}
ST_lyap	0.023 ± 0.029	0.027 ± 0.029	0.140	1.29×10^{-5}	2.94×10^{-5}
T_len	4.72 ± 3.03	4.06 ± 3.00	-0.220	3.30×10^{-8}	1.06×10^{-7}
T_entropy	4.06 ± 0.40	4.01 ± 0.43	-0.121	2.11×10^{-2}	3.56×10^{-2}
T_energy	$6.42 \pm 7.21^{\dagger}$	$4.14 \pm 5.92^{\dagger}$	-0.347	4.86×10^{-16}	1.56×10^{-14}
T_lam1ratio	0.932 ± 0.080	0.909 ± 0.094	-0.269	6.72×10^{-10}	4.30×10^{-9}

† Energy values are scaled by 10^4 for display.

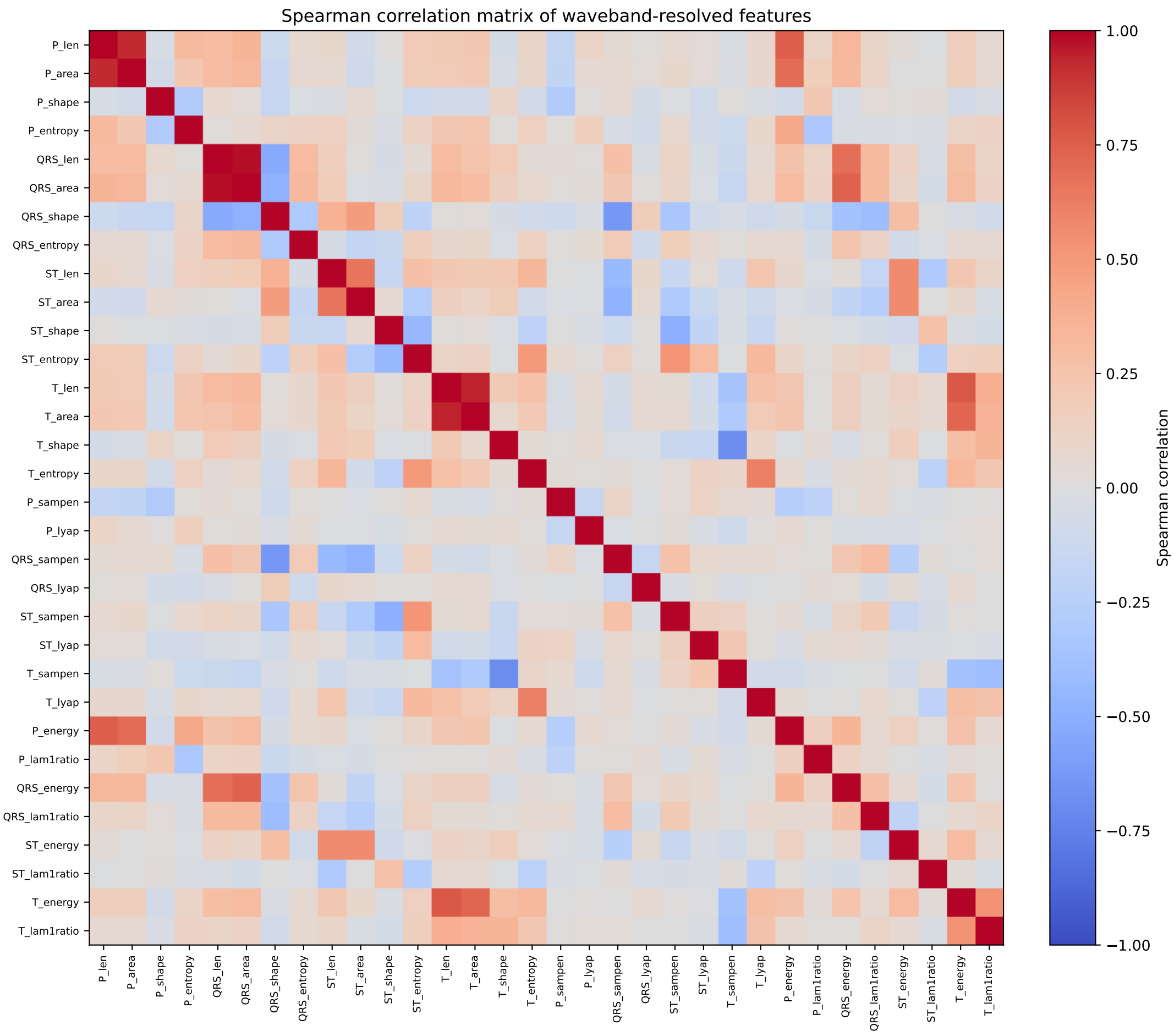

Supplementary Figure S3. Spearman correlation matrix of the extracted waveband-resolved features. Pairwise Spearman correlations were computed across the extracted waveband-resolved feature set to assess feature collinearity and descriptor redundancy. The matrix shows that some descriptors derived from related wavebands or feature categories exhibit moderate correlations, whereas the overall feature set does not collapse into a single redundant feature block. This analysis supports the interpretation of odds ratios as descriptive univariate association measures rather than independent multivariable predictors.

Virtual-Lead Construction and Spatial Averaging Rationale

The SERF–MCG array used in this study is sensitive to both cardiac magnetic-field activity and environmental or sensor-specific noise. In the present analysis, we constructed a global virtual lead by directly averaging all 36 channels after baseline correction and filtering.

This full-channel averaging strategy was used to improve global waveform stability and cross-subject comparability for phase-space reconstruction. At the same time, spatial averaging may attenuate localized magnetic-field patterns and high-spatial-frequency spatiotemporal information that could be relevant to ischemic territory localization. Therefore, the present analysis should be interpreted as a global virtual-lead analysis rather than a full channel-resolved spatiotemporal MCG mapping study. Future work should directly compare global virtual-lead features with channel-resolved and multi-channel spatiotemporal embeddings.
